# Uterine morphology and anomalies in women with and without polycystic ovary syndrome: a systematic review and meta-analysis

**DOI:** 10.1093/humrep/deaf117

**Published:** 2025-06-19

**Authors:** Lucja Zaborowska, Joanna Blok, Inga Ludwin, Jakub Jasek, Steven R Lindheim, Wellington P Martins, Artur Ludwin

**Affiliations:** Doctoral School of Medical and Health Sciences, Jagiellonian University Medical College, Kraków, Poland; 1st Department of Obstetrics and Gynecology, Medical University of Warsaw, Warsaw, Poland; 1st Department of Obstetrics and Gynecology, Medical University of Warsaw, Warsaw, Poland; Ludwin & Ludwin Private Medical Center, Kraków, Poland; Department of Gynecology and Oncology, Jagiellonian University Medical College, Kraków, Poland; 1st Department of Obstetrics and Gynecology, Medical University of Warsaw, Warsaw, Poland; Department of Obstetrics and Gynecology, Wright State University, Boonshoft School of Medicine, Dayton, OH, USA; SEMEAR fertilidade, Ribeirao Preto, Brazil; 1st Department of Obstetrics and Gynecology, Medical University of Warsaw, Warsaw, Poland; Ludwin & Ludwin Private Medical Center, Kraków, Poland

**Keywords:** uterine morphology, congenital uterine malformations, polycystic ovary syndrome, uterus, uterine anomalies, Mullerian anomalies

## Abstract

**STUDY QUESTION:**

Does polycystic ovary syndrome influence the morphology of the uterus and the prevalence of congenital uterine anomalies?

**SUMMARY ANSWER:**

Available evidence of very low quality shows a possible association between PCOS and some uterine anomalies.

**WHAT IS KNOWN ALREADY:**

Congenital uterine malformations range from subtle changes in uterine shapes to major morphologic deformities It is postulated that major anomalies result from the abnormal development of Mullerian ducts. However, the etiology of minor deformities is a subject of speculation. Many factors may impact the development of the uterus and its morphology *in utero* and after birth. Many researchers have reported on the increased prevalence of uterine anomalies in PCOS patients, yet the synthesis of evidence has not been performed.

**STUDY DESIGN, SIZE, DURATION:**

A systematic review and meta-analysis were performed. We systematically searched the PubMed, Scopus, Embase, and Cochrane Library databases using a complex search strategy including terms such as e.g. ‘uterus’, ‘polycystic ovary syndrome’, ‘morphology’, ‘dimensions’, ‘congenital uterine anomalies’, and their synonyms. No language or date restrictions were applied during the literature search. The meta-analysis was based on odds ratio (OR) and 95% CIs using the Mantel–Haenszel random effects model.

**PARTICIPANTS/MATERIALS, SETTING, METHODS:**

The primary outcomes included uterine malformations, dimensions, and volume, assessed using non-invasive and invasive methods in observational studies comparing PCOS and non-PCOS patients. We excluded case reports, case series, reviews, studies without control groups, and articles reporting exclusively on polycystic ovary morphology. The modified Newcastle–Ottawa scale was used to assess the quality of the included research.

**MAIN RESULTS AND THE ROLE OF CHANCE:**

Our search retrieved 1929 identified publications, 11 of which were included in the systematic review (10 581 women). The majority of the studies were of poor quality. PCOS was associated with higher prevalence of uterine anomalies (OR = 5.96; 95% CI = 1.22–29.17, *P* = 0.028). The general count of uterine malformations was significantly higher in the PCOS group (25.0%, 95% CI 6.9–43.2, range 0–49%) than in the non-PCOS group (5.3%, 95% CI 0.5–14.8, range 0–23.1%). The studies had a very high heterogeneity. The scope of uterine malformations possibly linked to PCOS included septate, didelphys, I-shaped (morphology that is not recognized by any main classifications), and dysmorphic uteri. The septate uterus was the most often reported malformation. Altered dimensions included fundal indentation depth and angle. Available research used four classification systems: the most frequently reported was that of the American Fertility Society 1988 (3/11). Six studies did not use any classification. The assessment methods included 2- and 3-dimensional ultrasound, magnetic resonance imagining, hysterosalpingography, laparoscopy, and hysteroscopy.

**LIMITATIONS, REASONS FOR CAUTION:**

Most of the available studies are of very low quality, use inadequate assessment methods and classifications, and are prone to a high risk of selection bias. Additionally, the differences between PCOS phenotypes and their hormonal aspects are poorly addressed and do not sufficiently explain the etiology of observed changes. The included research displays a varying level of heterogeneity.

**WIDER IMPLICATIONS OF THE FINDINGS:**

Further research is needed to (i) confirm whether the association between uterine anomalies and PCOS is clinically relevant, (ii) evaluate whether the prevalence of the uterine anomalies varies between women with different hormonal disturbances (e.g. hyperandrogenism), (iii) assess the benefit of any intervention for uterine anomalies in women with PCOS.

**STUDY FUNDING/COMPETING INTEREST(S):**

This study received no funding. The authors have no conflicts to disclose.

**REGISTRATION NUMBER:**

CRD42024527964; http://www.crd.york.ac.uk/prospero/

## Introduction

PCOS is the most common cause of subfertility worldwide (Azziz *et al.*, 2004), and may affect up to 13% of the population (Bozdag *et al.*, 2016). The disease varies in presentation and is mainly characterized by the coexistence of hyperandrogenism and disrupted ovulation, as diagnosed by the Rotterdam criteria (Rotterdam ESHRE/ASRM-Sponsored PCOS Consensus Workshop Group, 2004). PCOS occurs in women of reproductive age and may affect multiple members of the same family, suggesting a genetic background (Kahsar-Miller *et al.*, 2001; Vink *et al.*, 2006; Coviello *et al.*, 2011; Risal *et al.*, 2019). The associated morphological changes in the uterus in PCOS have only recently begun to be adequately examined and reported (Grimbizis *et al.*, 2013; Saravelos *et al.*, 2017; Ludwin *et al.*, 2019, 2020; Pfeifer *et al.*, 2021).

Isolated uterine anomalies have been linked to recurrent miscarriages, preterm labor, infertility, and the increased use of assisted reproductive technology (Saravelos *et al.*, 2008; Jayaprakasan *et al.*, 2011). Various factors may disturb Müllerian duct development and lead to different fusion defects (Kaufman *et al.*, 1977; Roly *et al.*, 2018). PCOS is characterized by excessive exposure to androgens and anti-Müllerian hormone (Filippou and Homburg, 2017; Piltonen *et al.*, 2019); these have been linked to disturbance of HOX gene, uncovering a possible relationship between uterine malformations and PCOS (de Zegher *et al.*, 1998; Cheng *et al.*, 2011; Tata *et al.*, 2018; Aslan *et al.*, 2022; Fujii and Oguchi, 2023). The final size and shape of the uterus depend on the reproductive stage and the influence of sex hormones; therefore, we cannot exclude the potential impact of prolonged hormonal imbalance in the formation of minor outliers. In fact, even microanomalies of the uterine structure may play a significant role in the clinical spectrum of the disease and may aggravate the infertility struggles of affected women.

In this systematic review, we aim to:

I. Assess the current state of knowledge on the prevalence of congenital uterine anomalies in the setting of PCOS. Specifically, our goal was to properly assess the morphological forms of the uterus in PCOS in accordance with the latest definitions of norms and outliers. Identifying susceptible groups that would possibly benefit from early surgical treatment was one of our main objectives.
II. Summarize the most important aspects of PCOS-related changes in uterine morphology and their potential clinical significance. The article discusses clinical trials focused on potential features of PCOS within the uterus.
III. Identify the gaps in current evidence to suggest new avenues for further research.

## Materials and methods

The protocol of this systematic review was registered with PROSPERO (registration number: CRD42024527964; http://www.crd.york.ac.uk/prospero/). Our manuscript followed the Preferred Reporting Items for Systematic Reviews and Meta Analyses (PRISMA) 2020 reporting guidelines (Page *et al.*, 2021).

### Eligibility criteria

The primary outcomes included uterine measurements and morphology in PCOS patients in clinical settings. The investigated topics included uterine congenital malformations, uterine longest diameter (ULD), uterine volume (UV), myometrial thickness, fundal indentation depth, and angle, assessed using non-invasive and invasive methods in non-pregnant women. We included research irrespectively of its objective, control group characteristics, classifications, study type (cohort, cross-sectional, case-control, prospective or retrospective), or diagnostic methods used. We excluded case reports, case series, reviews, studies without control groups, and articles reporting exclusively on polycystic ovary morphology.

### Search strategy

The review was performed by searching the PubMed, Scopus, Embase, and Cochrane Library electronic databases. There were no restrictions on date or status of the publication. The last electronic search for each database was performed on 3 March 2024. The basic PubMed search string and keywords used are available in Supplementary Data File S1. The search was performed simultaneously by two authors (L.Z., J.B.), and was modified according to the technical requirements of each database. Although no language restrictions were applied during the literature search, we did use English keywords. All articles were initially screened for their titles and abstracts. Additionally, the authors manually searched the reference lists of the included articles.

The full-text versions of included publications were retrieved by two authors (L.Z., J.B.) with the help of librarians of the institution of the first author.

### Data collection and extraction

Full-text analysis and data extraction were performed in a standardized way by four authors (L.Z. and I.L., J.B. and J.J.) working in pairs. Any potential disagreements within a pair of co-authors were resolved by a consultation with a fifth author (A.L.). The extracted data tackled the following subjects: study type, types of uterine malformations assessed with different international classifications, prevalence of uterine malformations, methods of assessment, population characteristic, study group characteristics, and suspected etiology of observed changes.

### Quality assessment of studies

The modified Newcastle–Ottawa scale was used to assess the quality of the non-randomized studies included in the quantitative summaries (Stang, 2010; Mata *et al.*, 2015). The scale consists of different domains, such as selection, comparability, outcomes, and statistics. Studies were divided between the low-risk-of-bias group (>6 points on the modified Newcastle–Ottawa scale) and the high-risk-of-bias group (≤6 points). Full details of the scoring method are available in Supplementary Data File S2. The assessment was independently performed by two authors (L.Z., J.B.); any discrepancies were resolved by discussion, and all disagreements were reviewed by a third researcher (A.L.).

### Synthesis of results

The quantitative summary was performed separately for studies regarding uterine malformations and uterine measurements of PCOS patients in clinical settings. Due to the heterogeneity of the studies included, the synthesis was performed solely for the prevalence of uterine anomalies. The PCOS subjects and controls were compared based on odds ratio (OR) and 95% CIs, using Mantel–Haenszel random effects model. A *P*-value of ≤0.05 was considered significant. *I*^2^ statistics were calculated to provide a measure of the proportion of overall variation attributable to between-study heterogeneity. We performed sub-analyses for each type of reported congenital malformation. The statistical analyses were performed using PythonMeta module ver. 1.26 (rel. 17.11.2021).

## Results

### Study selection and characteristics

**Figure 1.**
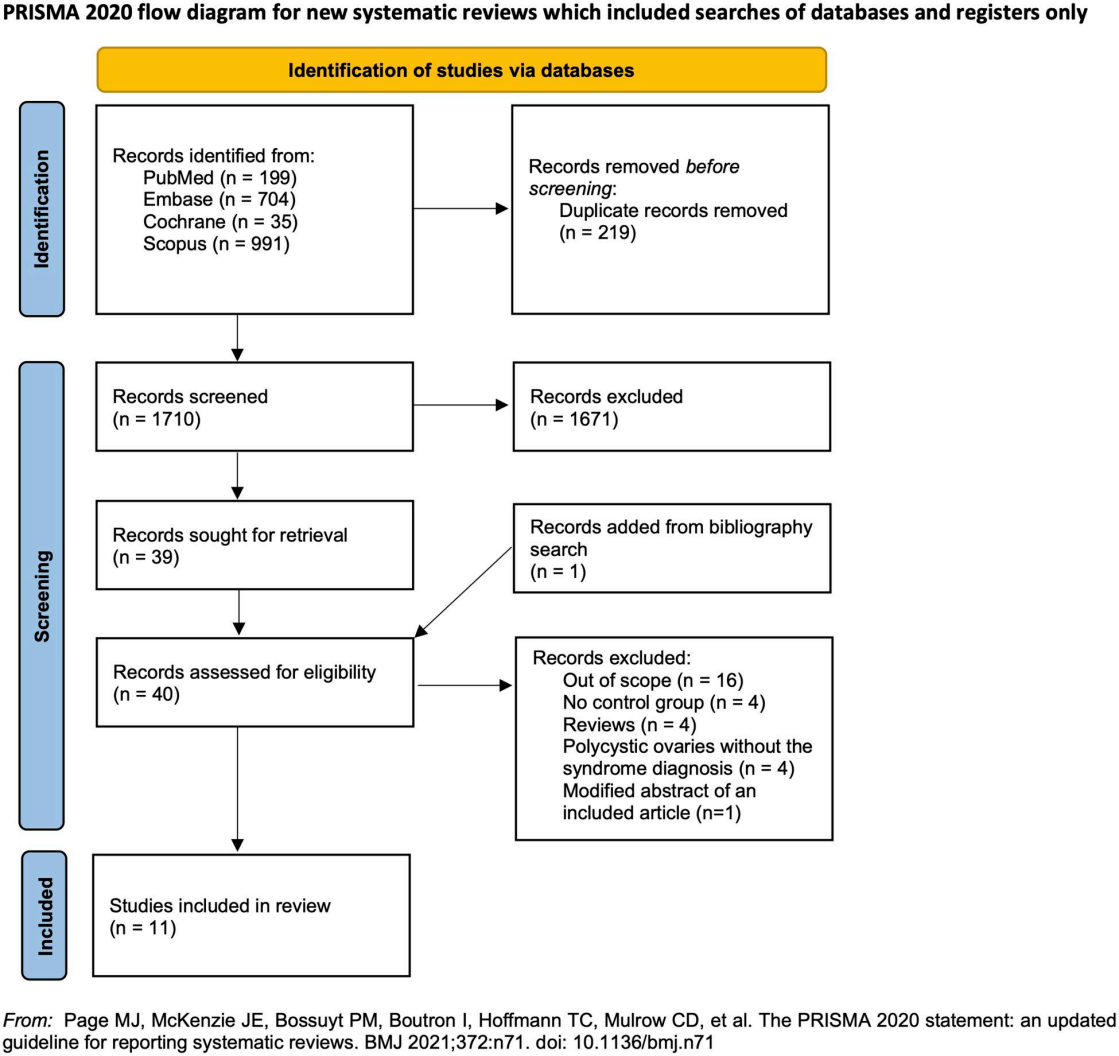
**PRISMA flowchart of included studies**.

A total of 1929 publications were identified from different databases (PubMed = 199, Embase = 704, Cochrane = 35, Scopus = 991). A total of 219 duplicated records were removed either by automatic or manual search. After screening titles/abstracts, we excluded 1671 articles that did not meet our inclusion criteria. One additional study was identified by search of the reference list (Moramezi *et al.*, 2013). We evaluated 40 potentially eligible records in full text available, 11 of which were included in the systematic review (Fig. 1, Supplementary Data File S3). We included 10 full articles and 1 abstract.

Regarding the study design, 5/11 of the studies were described by the original authors as prospective (observational, case–control, cohort), 3/11 as retrospective (cohort, case-control), 1/11 as prospective-retrospective (cohort), while two were categorized only as a case–control and cross-sectional studies, respectively. The study types of eight publications were classified differently by the current study authors, resulting in a significant proportion of cross-sectional studies (8/11) (Table 1). Six studies were further re-assessed as prospective, three as retrospective, one as prospective-retrospective (Table 1).

**Table 1.** Main characteristics of included studies.

Article	**Study type** ^a^	Population	Age, years*	PCOS diagnosis	Uterine anomaly classification	Method of assessment	Reported morphologies
Fujii and Oguchi (2023)	Cross-sectional, prospective	333 women with subfertility	32.8 ± 0.5 (PCOS); 33.8 ± 0.3 (Controls)	2003 Rotterdam criteria	ASRM MAC 2021	3D TVS	Arcuate uterus, Septate uterus, Myometrial thickness, Fundal indentation depth, Fundal indentation angle
Aslan *et al.* (2022)	Case–control, retrospective	103 PCOS women without infertility. Controls were women with infertility due to male factor.	31.8 ± 4.4 (PCOS); 31.9 ± 5 (Controls)	2003 Rotterdam criteria	AFS/ASRM 1988, ESHRE/ESGE	HSG	Arcuate uterus, Septate uterus, Dysmorphic^b^
Tokhunts *et al.* (2022)	Cross-sectional, retrospective and prospective	289 women with subfertility or recurrent pregnancy loss	25.2 ± 3.5	2003 Rotterdam criteria	ESHRE/ESGE and CUME 2019	3D TVS	Septate uterus, I-shaped uterus, T-shaped uterus, Bicornuate uterus, Unicornuate uterus
Albdairi and Al-Shalah (2021)	Cross-sectional, retrospective	1374 women with subfertility	32.4 ± 7.0 (with septum); 31.7 ± 7.2 (without septum)	2003 Rotterdam criteria	Not reported	TVS, HSG, laparoscopy, hysteroscopy, MRI	Septate uterus
Ege *et al.* (2019)	Cross-sectional, retrospective	3033 women with subfertility	Not reported	2003 Rotterdam criteria	AFS/ASRM 1988	TVS, HSG, laparoscopy, hysteroscopy, MRI	Arcuate uterus, Septate uterus, T-shaped uterus, Bicornuate uterus, Didelphys uterus, Unicornuate uterus
Panidis *et al.* (2014)	Cross-sectional, prospective	1198 women without infertility	23.7 ± 5.3 (PCOS); 30.5 ± 5.5 (Controls)	2003 Rotterdam criteria	Not applicable	TVS	Uterine volume
Saleh and Shawky Moiety (2014)	Cross-sectional, prospective	3900 women with subfertility	27.9 ± 4.9	2003 Rotterdam criteria	AFS/ASRM 1988	3D TVS, HSG, laparoscopy, hysteroscopy, MRI	Arcuate uterus, Septate uterus, T-shaped uterus, Bicornuate uterus, Didelphys uterus, Unicornuate uterus
Moramezi *et al.* (2013)	Case–control	166 women without infertility	27.1 ± 4.7 (PCOS); 30.0 ± 6.1 (Controls)	Polycystic ovaries, oligomenorrhea, hirsutism	Not reported	2D TVS, SIS	Arcuate uterus, Septate uterus
Leonhardt *et al.* (2012)	Case–control, prospective	83 women without infertility	29.5 ± 4.5 (PCOS); 27.6 ± 3.2 (Controls)	2003 Rotterdam criteria	Not reported	3D TVS, MRI	Myometrial thickness, General count of uterine anomalies
Kawano *et al.* (1987)	Cross-sectional, prospective	33 women without infertility	Not reported	Polycystic ovaries, sterility/irregular menses, hormonal analysis (LH and FSH levels)	Not applicable	Not applicable	Uterine volume, Uterine longest diameter
Orsini *et al.* (1985)	Cross-sectional, prospective	69 women without infertility	15–36 (PCOS); 16–38 (Controls)	Polycystic ovaries, oligomenorrhea/amenorrhea, obesity, hirsutism, hyperandrogenism, abnormal gonadotropin secretion	Not applicable	Not applicable	Uterine volume

a As assessed by the current study authors.

b Uterine morphology described as ESHRE-ESGE U1c/ASRM VII by the study authors.

* Mean ± SD or range (min–max).

AFS, American Fertility Society; ASRM, American Society for Reproductive Medicine; CUME, Congenital Uterine Malformation by Experts; ESGE, European Society for Gynaecological Endoscopy; HSG, hysterosalpingography; MAC, Müllerian Anomalies Classification; PCOS, polycystic ovary syndrome; SIS, sonohysterography; TVS, transvaginal ultrasound.

### Population characteristics

A total of 10 581 women were evaluated by the included studies, with 9281 subjects assessed for uterine malformations. The total number of PCOS patients included was 2940. Control groups encompassed healthy subjects (5/11 studies) and infertility patients (6/11 studies, including 1 male infertility) (Tables 2 and 4). Included PCOS patients were reported as infertile in 5/11 studies. Overall, the non-PCOS patients had significantly higher infertility prevalence (95.7%, 7311/7643) than the PCOS group (56.8%, 1670/2938; χ^2^(1) = 2491.10, *P* < 0.001). Studies that directly reported on the prevalence of uterine malformations were more prone to use infertility patients in PCOS (5/8) and control groups (6/8). Despite concomitant hyperandrogenism, we decided to exclude patients with congenital adrenal hyperplasia described in one of the articles (Tokhunts *et al.*, 2022).

**Table 2.** Uterine morphology in PCOS and non-PCOS patients.

General morphology	Conclusions	Population	Number of anomalies in a PCOS group (%) vs non-PCOS group (%)	*P-*value	Classification	Authors
All uterine anomalies	Increased prevalence in PCOS patients	103 patients (51 PCOS vs 52 male factor infertility)	25/51 (49.0) vs 12/52 (23.1)	*P*<0.01	AFS/ASRM 1988	Aslan *et al.* (2022)
			17/51 (33.3) vs 3/52 (5.8)	*P*<0.01	ESHRE/ESGE	
		3033 patients (710 PCOS vs 2323 infertility)	57/710 (8) vs 74/2323 (3.2)	*P*<0.001	AFS/ASRM 1988	Ege *et al.* (2019)
		3900 patients (409 PCOS vs 3491 infertility)	149/409 (36.4) vs 55/3491 (1.6)	*P*<0.001	AFS/ASRM 1988	Saleh and Shawky (2014)
		166 patients (83 PCOS vs 83 healthy subjects)	29/83 (34.9) vs 10/83 (12.0)	*P*=0.001	Not reported	Moramezi *et al.* (2013)
	No difference between PCOS/control	83 patients (55 PCOS vs 28 healthy subjects)	0/55 (0) vs 0/28 (0)	*P*>0.05	Not reported	Leonhardt *et al.* (2012)
	Total: 7285 patients (1308 PCOS vs 5977 control)					
Arcuate uterus	Increased prevalence in PCOS patients	333 patients (93 PCOS vs 240 infertility)	Not reported	Not reported	ASRM MAC 2021	Fujii and Oguchi (2023)
		3033 patients (710 PCOS vs 2323 infertility)	30/710 (4.2) vs 45/2323 (1.9)	*P*=0.003	AFS/ASRM 1988	Ege *et al.* (2019)
		3900 patients (409 PCOS vs 3491 infertility)	99/409 (24.2) vs 37/3491 (1.1)	*P*<0.00001	AFS/ASRM 1988	Saleh and Shawky (2014)
	No difference between PCOS/control	103 patients (51 PCOS vs 52 male factor infertility)	8/51 (16) vs 9/52 (17.3)	*P*=0.82	AFS/ASRM 1988	Aslan *et al.* (2022)
		166 patients (83 PCOS vs 83 healthy subjects)	6/83 (7.2) vs 4/83 (4.8)	*P*=0.514	Not reported	Moramezi *et al.* (2013)
	Total (available data): 7202 patients (1253 PCOS vs 5949 control)					
Septate uterus	Increased prevalence in PCOS patients	333 patients (93 PCOS vs 240 infertility)	3/93 (3.2) vs 0/240 (0)	*P*=0.02	ASRM MAC 2021	Fujii and Oguchi (2023)
		103 patients (51 PCOS vs 52 male factor infertility)	15/51 (29.4) vs 3/52 (5.8)	*P*<0.01	AFS/ASRM 1988 and ESHRE/ESGE	Aslan *et al.* (2022)
		1374 patients (384 PCOS vs 990 infertility)	219/384 (57.0) vs 468/990 (47.3)	*P*=0.001	Not reported	Albdairi and Al-Shalah (2021)
		3033 patients (710 PCOS vs 2323 infertility)	19/710 (2.7) vs 13/2323 (0.6)	*P*<0.001	AFS/ASRM 1988	Ege *et al.* (2019)
		3900 patients (409 PCOS vs 3491 infertility)	34/409 (8.3) vs 14/3491 (0.4)	*P*<0.00001	AFS/ASRM 1988	Saleh and Shawky (2014)
		166 patients (83 PCOS vs 83 healthy subjects)	23/83 (27.7) vs 6/83 (7.2)	*P*=0.001	Not reported	Moramezi *et al.* (2013)
	No difference between PCOS/controls	289 patients (74 PCOS vs 215 infertility or habitual pregnancy loss)	3/74 (4.1) PCOS vs 19/215 (8.8)	Not reported	ESHRE/ESGE and CUME 2019	Tokhunts *et al.* (2022)
	Total: 9198 patients (1804 PCOS vs 7394 control)					
I-shaped uterus^a^	Increased prevalence in PCOS patients	289 patients (74 PCOS vs 215 infertility or habitual pregnancy loss)	18/74 (24.3) PCOS vs 0/215 (0)	*P*<0.5	ESHRE/ESGE and CUME 2019	Tokhunts *et al.* (2022)
T-shaped uterus	No difference between PCOS/controls	289 patients (74 PCOS vs 215 infertility or habitual pregnancy loss)	0/74 (0) PCOS vs 10/215 (4.7)	Not reported	ESHRE/ESGE and CUME 2019	Tokhunts *et al.* (2022)
		3033 patients (710 PCOS vs 2323 infertility)	0/710 (0) vs 2/2323 (0.1)	*P*=0.4	AFS/ASRM 1988	Ege *et al.* (2019)
		3900 patients (409 PCOS vs 3491 infertility)	1/409 (0.2) vs 0/3491 (0)	*P*=0.1049	AFS/ASRM 1988	Saleh and Shawky (2014)
	Total: 7222 patients (1193 PCOS vs 6029 control)					
Dysmorphic uterus^b^	Increased prevalence in PCOS patients	103 patients (51 PCOS vs 52 male factor infertility)	2/51 (3.9) vs 0/52 (0)	*P*<0.01	AFS/ASRM 1988 and ESHRE/ESGE	Aslan *et al.* (2022)
Bicornuate uterus	Increased prevalence in PCOS patients	3900 patients (409 PCOS vs 3491 infertility)	4/409 (1.0) vs 2/3491 (0.1)	*P*=0.0015	AFS/ASRM 1988	Saleh and Shawky (2014)
	No difference between PCOS/control	3033 patients (710 PCOS vs 2323 infertility)	3/710 (0.4) vs 7/2323 (0.3)	*P*=0.6	AFS/ASRM 1988	Ege *et al.* (2019)
		289 patients (74 PCOS vs 215 infertility or habitual pregnancy loss)	0/74 (0) PCOS vs 1/215 (0.47)	Not reported	ESHRE/ESGE and CUME 2019	Tokhunts *et al.* (2022)
	Total: 7222 patients (1193 PCOS vs 6029 control)					
Didelphys uterus	No difference between PCOS/control	3033 patients (710 PCOS vs 2323 infertility)	2/710 (0.3) vs 1/2323 (0)	*P*=0.07	AFS/ASRM 1988	Ege *et al.* (2019)
		3900 patients (409 PCOS vs 3491 infertility)	1/409 (0.2) vs 0/3491 (0)	*P*=0.1049	AFS/ASRM 1988	Saleh and Shawky (2014)
	Total: 6933 patients (1119 PCOS vs 5814 control)					
Unicornuate uterus	Increased prevalence in PCOS patients	3900 patients (409 PCOS vs 3491 infertility)	5/409 (1.2) vs 1/3491 (0)	*P*=0.0001	AFS/ASRM 1988	Saleh and Shawky (2014)
	No difference between PCOS/control	3033 patients (710 PCOS vs 2323 infertility)	3/710 (0.4) vs 4/2323 (0.2)	*P*=0.2	AFS/ASRM 1988	Ege *et al.* (2019)
		289 patients (74 PCOS vs 215 infertility or habitual pregnancy loss)	0/74 (0) PCOS vs 2/215 (0.9)	Not reported	ESHRE/ESGE and CUME 2019	Tokhunts *et al.* (2022)
	Total: 7222 patients (1193 PCOS vs 6029 control)					

a A subclass of uterine morphology that is not recognized by any major classification.

b Uterine morphology described as ESHRE-ESGE U1c/ASRM VII by the study authors.

AFS, American Fertility Society; ASRM, American Society for Reproductive Medicine; CUME, Congenital Uterine Malformation by Experts; ESGE, European Society for Gynaecological Endoscopy; MAC, Müllerian Anomalies Classification; PCOS, polycystic ovary syndrome.

### Assessment techniques

The included articles utilized both invasive and non-invasive techniques to assess the presence of uterine malformations (Table 1). Only four studies (Leonhardt *et al.*, 2012; Saleh and Shawky Moiety, 2014; Tokhunts *et al.*, 2022; Fujii and Oguchi, 2023) have used 3-dimensional ultrasonography as a reliable and accurate tool for diagnosing uterine malformations in the setting of PCOS (Ludwin *et al.*, 2013; Ludwin and Ludwin, 2016; Saravelos *et al.*, 2017). Four researchers performed magnetic resonance imagining for diagnosis confirmation (Leonhardt *et al.*, 2012; Saleh and Shawky Moiety, 2014; Ege *et al.*, 2019; Albdairi and Al-Shalah, 2021). Most included research utilized 2-dimensional ultrasonography, saline infusion sonography, hysterosalpingography, hysteroscopy, laparoscopy, or a combination of these methods (Moramezi *et al.*, 2013; Aslan *et al.*, 2022) (Table 1). Two-dimensional ultrasonography is characterized by unknown and limited accuracy in assessing minor and major uterine malformations, respectively (Saravelos *et al.*, 2008; Ludwin *et al.*, 2013). Its reliability in the proper classification of uterine anomalies is doubtful. Moreover, both hysteroscopy and laparoscopy exhibit very poor reliability in differentiating between basic uterine morphologies, i.e. normal, arcuate, and septate uterus (Smit *et al.*, 2013, 2015, 2016; Ludwin and Ludwin, 2016). It is worth noting that these minimally invasive methods may cause significant discomfort to affected patients(Chapron, 2002; Aas‐Eng *et al.*, 2017).

### Uterine malformations classifications

Included studies utilized a variety of classifications: American Fertility Society 1988—AFS 1988 (The American Fertility Society, 1988) (3/11), American Society for Reproductive Medicine Müllerian Anomalies Classification 2021—ASRM MAC 2021 (Pfeifer *et al.*, 2021) (1/11), the ESHRE and the European Society for Gynaecological Endoscopy (ESGE)—ESHRE/ESGE (Grimbizis *et al.*, 2013, 2016) (2/11) and Congenital Uterine Malformation by Experts 2019 (Ludwin *et al.*, 2018, 2020) (1/11). More than half of included studies did not cite any system at all (6/11) (Orsini *et al.*, 1985; Kawano *et al.*, 1987; Appelman *et al.*, 2003; Leonhardt *et al.*, 2012; Moramezi *et al.*, 2013; Panidis *et al.*, 2014). Two authors decided on the simultaneous use of two classifications (2/11), while three used only one system (3/11) (Table 1).

### Prevalence of uterine malformations

Out of 11 included studies, 8 directly reported on prevalence of uterine malformations, comparing patients with PCOS to different control groups (Table 2). All studies but one (Leonhardt *et al.*, 2012) included in this part of the review reported a statistically higher prevalence of uterine anomalies among the PCOS population (Moramezi *et al.*, 2013; Saleh and Shawky Moiety, 2014; Ege *et al.*, 2019; Albdairi and Al-Shalah, 2021; Aslan *et al.*, 2022; Tokhunts *et al.*, 2022; Fujii and Oguchi, 2023). Overall, the general prevalence of uterine anomalies was significantly higher in the PCOS group (25.0%, 95% CI 6.9–43.2, range 0–49%) than in the non-PCOS group (5.3%, 95% CI 0.5–14.8, range 0–23.1%) (Tables 2 and 3). PCOS was related to a greater probability of uterine anomalies compared to healthy, infertile, and non-PCOS women, based on the calculated odds ratio (Supplementary Data File S4). Using the Mantel–Haenszel random effects model, the observed result was statistically significant OR = 5.96 (95% CI = 1.22–29.17, *P* = 0.028). In both analyses, we identified very high heterogeneity across studies (*I*^2^ = 97.56%), further downgrading the quality of the evidence based on observational research (Supplementary Data File S4).

**Table 3.** Prevalence of uterine anomalies in PCOS and non-PCOS patients.

Uterine morphology	No. of studies	Prevalence of uterine malformations, % (95% CI)		*P*-value^a^
		PCOS	non-PCOS	
All uterine anomalies	5	25.0 (6.9–43.2)	5.3 (0.5–14.8)	0.028
Arcuate uterus	5	12.6 (3.3–21.9)	4.4 (0.6–11.4)	0.211
Septate uterus	7	18.7 (3.5–33.9)	5.9 (0.3–17.9)	0.009
I-shaped uterus^c^	1	24.3^b^	0.0^b^	<0.05^b^
T-shaped uterus	3	0.04 (0–0.3)	0.6 (0–4.3)	0.9
Dysmorphic uterus^d^	1	3.9^b^	0.0^b^	<0.01^b^
Bicornuate uterus	3	0.5 (0.2–1.1)	0.2 (0.03–0.5)	0.222
Didelphys uterus	2	0.3 (0.05–0.7)	0.0001 (0–0.09)	0.016
Unicornuate uterus	3	0.6 (0.09–1.4)	0.2 (0.004–2.1)	0.199

a Comparisons of ≥2 studies were made using meta-analysis.

b The single study included; data reported by the study authors.

c A subclass of uterine morphology that is not recognized by any major classification.

d Uterine morphology described as ESHRE-ESGE U1c/ASRM VII by the study authors.

PCOS, polycystic ovary syndrome.

**Figure 2.**
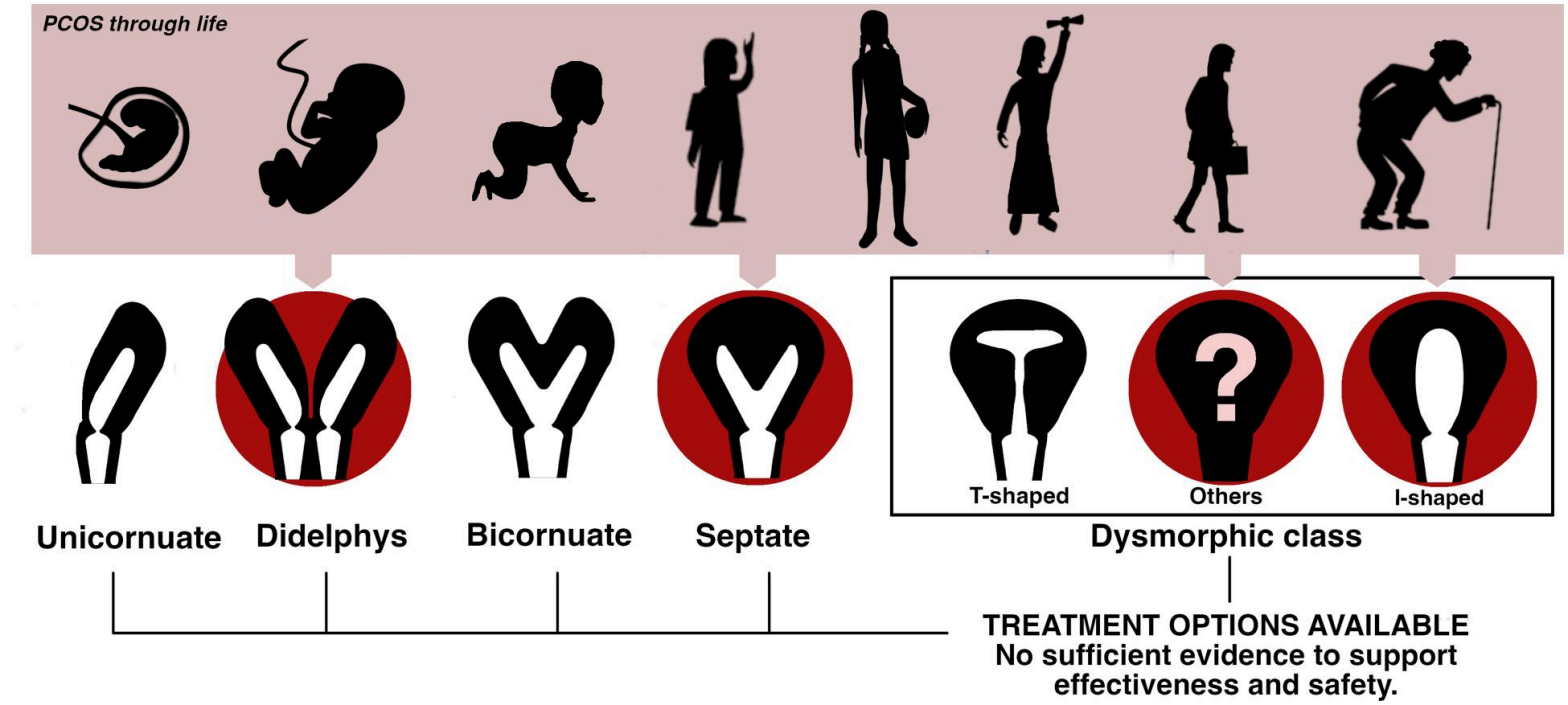
**Uterine anomalies observed more frequently in a PCOS setting.** A graphical representation of the study results. Very low-quality evidence shows a possible association between PCOS and the morphologies of didelphys, septate, I-shaped, and dysmorphic uteri (encircled in red). Described differences may arise from disturbances in intra-uterine development or hormonal imbalances experienced later in life.

**Figure 3.**
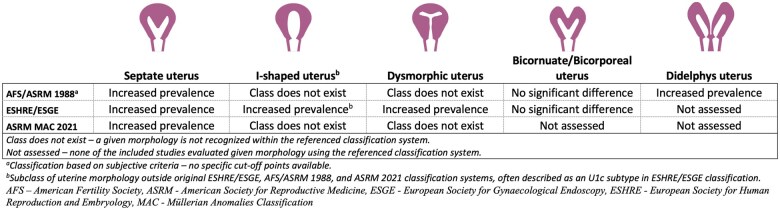
**PCOS-based prevalence of uterine morphologies according to international criteria**.

Among separately assessed uterine malformations, the possible association and increased prevalence in PCOS patients were found for septate, didelphys, I-shaped, and dysmorphic uteri (Moramezi *et al.*, 2013; Saleh and Shawky Moiety, 2014; Ege *et al.*, 2019; Aslan *et al.*, 2022; Fujii and Oguchi, 2023) (Figs 2 and 3). The most frequent conclusion was the significantly higher presence of a uterine septum in the PCOS group (18.7%, 95% CI 3.5–33.9) than in the control group (5.9%, 95% CI 0.3–17.9) (Table 3).

The presence of arcuate and septate uterus was assessed by five and seven articles, respectively. The increased prevalence of specific malformation was reported in 3/5 articles regarding the arcuate uterus, and 6/7 articles regarding the septate uterus. The performed meta-analysis indicated a statistically significant association with PCOS only in the case of septate uterus (OR = 4.21, 95% CI 1.44–12.31, *P* = 0.009; Table 3, Supplementary Data File S4). The differences in prevalence of arcuate uterus were found to be non-significant (OR = 3.22, 95% CI 0.52–20.06, *P* = 0.211; Table 3, Supplementary Data File S4). Data regarding the arcuate uterus were assessed using only American classifications (ASRM MAC 2021, AFS/ASRM 1988) (The American Fertility Society, 1988; Pfeifer *et al.*, 2021), as the ESHRE/ESGE system does not recognize this morphology, which is often considered a variant of the norm (Grimbizis *et al.*, 2013; Ludwin and Ludwin, 2015, 2016). In both cases, the heterogeneity between included studies was very high (Supplementary Data File S4).

Only one article described the increased prevalence of dysmorphic uterus (Aslan *et al.*, 2022), a category present in the ESHRE/ESGE classification (Grimbizis *et al.*, 2013, 2016). Interestingly, some other authors decided to evaluate separate subgroups of dysmorphic uteri, namely T-shaped and I-shaped uterus.

No statistical association was found with the prevalence of T-shaped uterus, assessed in three articles (*P* = 0.9; Table 3, Supplementary Data File S4) (Saleh and Shawky Moiety, 2014; Ege *et al.*, 2019; Tokhunts *et al.*, 2022). Only one research (Tokhunts *et al.*, 2022) reported on increased prevalence of an I-shaped uterus, an anomaly that is not widely recognized by any of the mainstream classification systems (The American Fertility Society, 1988; Grimbizis *et al.*, 2013, 2016; Ludwin *et al.*, 2018, 2020; Pfeifer *et al.*, 2021).

Bicornuate, didelphys, and unicornuate uteri were described by three, two, and three articles, respectively (Saleh and Shawky Moiety, 2014; Ege *et al.*, 2019; Tokhunts *et al.*, 2022). A significantly greater probability of uterine anomalies in PCOS patients was found only in the cases of didelphys uteri (OR = 10.72, 95% CI 1.57–73.20, *P* = 0.016). In this case, the heterogeneity between studies was low (*I*^2^ of 0%) (Supplementary Data File S4). The prevalences of bicornuate and unicornuate uterus were found to be non-significant with a *P*-value of 0.222 and 0.199, respectively (Table 2, Supplementary Data File S4).

Detailed results of all meta-analyses can be found in Supplementary Data File S4.

### Uterine measurements in PCOS

Out of 11 included studies, 5 reported on dimensions and measurements of uterus in PCOS, as compared with different control subjects (Table 4). Reported measurements included ULD, UV, myometrial thickness, fundal indentation depth, and fundal indentation angle (Orsini *et al.*, 1985; Kawano *et al.*, 1987; Leonhardt *et al.*, 2012; Panidis *et al.*, 2014; Fujii and Oguchi, 2023).

**Table 4.** Uterine measurements in PCOS and non-PCOS patients.

Ultrasound uterine measurements	Conclusions	Population	Values in PCOS patients vs in non-PCOS patients	*P*-value	Authors
Uterine longest diameter	No difference between PCOS/healthy patients	33 patients (19 PCOS vs 14 healthy control)	Data not available	Not significant	Kawano *et al.* (1987)
Uterine volume	No difference between PCOS/healthy patients^a^	1198 patients (1016 PCOS vs 182 healthy controls)	Mean ± SD (cm^3^) 39.0 ± 18.8 vs 41.6 ± 20.2	Not significant	Panidis *et al.* (2014)
		33 patients (19 PCOS vs 14 healthy controls)	Data not available	Not significant	Kawano *et al.* (1987)
	Reduced in PCOS patients	69 patients (44 PCOS vs 25 healthy controls)	Mean ± SD (cm^3^) 52.51 ± 20.54 vs 69.18 ± 26.62	*P*<0.01	Orsini *et al.* (1985)
Myometrial thickness	No difference between PCOS/healthy patients	83 patients (55 PCOS vs 28 healthy controls)	Mean ± SD (mm) 3D TVS 13.3 ± 3.7 vs 13.1 ± 2.1 MRI 14.1 ± 3.0 vs 14.5 ± 1.9	*P*=0.564^b^ *P*=0.242^b^	Leonhardt *et al.* (2012)
	Reduced in PCOS patients	333 patients (93 PCOS vs 240 infertility)	Mean ± SE (mm) 10.8 ± 0.2 vs 11.4 ± 0.2	*P*=0.0322	Fujii and Oguchi (2023)
Fundal indentation depth	Increased in PCOS patients	333 patients (93 PCOS vs 240 infertility)	Mean ± SE (mm) 2.2 ± 0.4 vs 0.0 ± 0.2	*P*<0.0001	Fujii and Oguchi (2023)
Fundal indentation angle	More acute in PCOS patients	333 patients (93 PCOS vs 240 infertility)	Mean ± SE (deg) 162.9 ± 2.2 vs 175.2 ± 1.3	*P*<0.0001	Fujii and Oguchi (2023)

a Panidis *et al.* found that patients with classic PCOS phenotypes diagnosed with the NIH criteria had smaller uterine volume than patients diagnosed with additional phenotypes introduced with the Rotterdam criteria.

b Adjusted *P*-value (age, and BMI and uterine measurements).

PCOS, polycystic ovary syndrome; TVS, transvaginal ultrasound.

**Figure 4.**
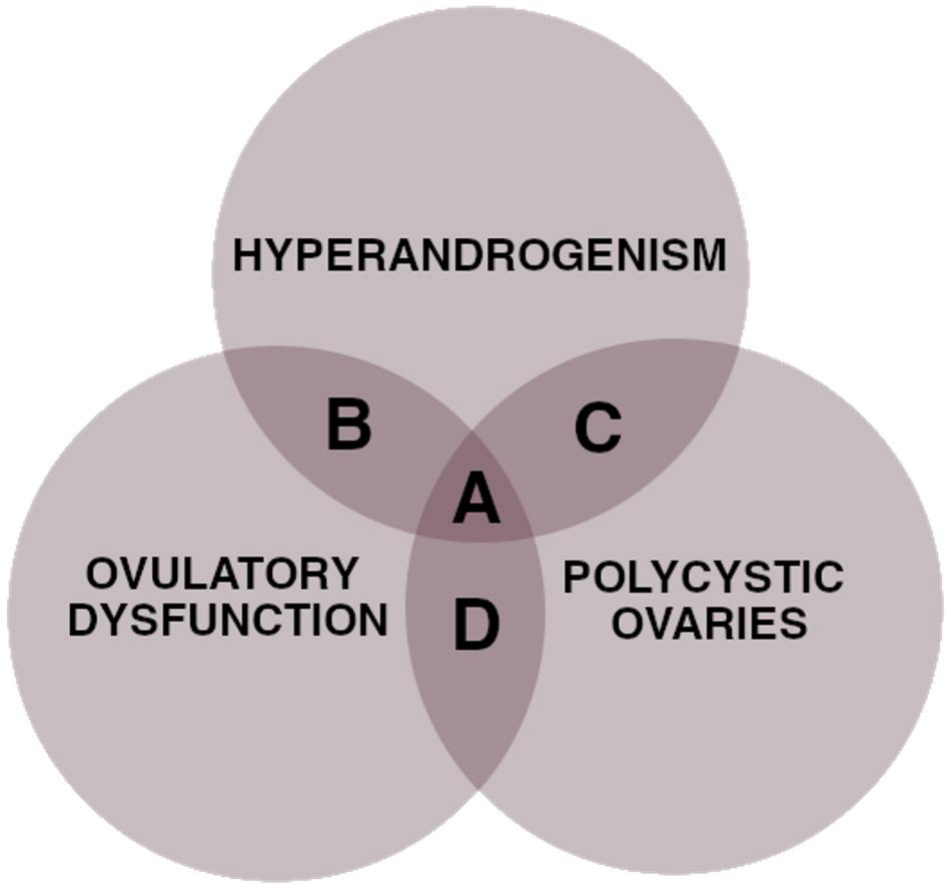
**Phenotypes of PCOS.** (**A** and **B**) ‘Classic’ PCOS phenotypes. (**C** and **D**) Phenotypes based on polycystic ovarian morphology and hyperandrogenism/oligo-ovulation.

Reported data have generally not shown statistically significant differences in ULD and UV in PCOS patients when compared to healthy subjects. However, one prospective study (Panidis *et al.*, 2014) with a considerably large population (1198 patients) showed a positive correlation between age, obesity, and UV in PCOS patients aged  < 40 years (Panidis *et al.*, 2014). Among the study group, women with ‘classic’ PCOS phenotypes (Fig. 4A and B) had smaller uterine cavities than women with phenotypes based on polycystic ovarian morphology and hyperandrogenism/oligo-ovulation (Fig. 4C and D) (Panidis *et al.*, 2014). Only one study (Fujii and Oguchi, 2023) reported on the internal indentation depth and angle—both of these measurements were found to be significantly altered in PCOS patients (Table 4). These findings are consistent with increased prevalence of arcuate and septate uterus, reported by the same authors (Fujii and Oguchi, 2023).

Two studies reported on the myometrial thickness of the uterus (Leonhardt *et al.*, 2012; Fujii and Oguchi, 2023), with unconclusive results (Table 4). The evidence on what constitutes a normal, thin, or thick myometrium in the reproductive age is limited (Traiman *et al.*, 1996). However, women of childbearing age typically have a uterine wall thickness of less than 20 mm (Traiman *et al.*, 1996; Youm *et al.*, 2011), and this value varies in different regions of the uterus (Ludwin *et al.*, 2014, 2015; Knez *et al.*, 2018). This problem is poorly recognized in studies focused on myometrial thickness.

### Quality of studies

Out of 11 studies included in the review, 8 were considered to be of poor quality (Orsini *et al.*, 1985; Kawano *et al.*, 1987; Moramezi *et al.*, 2013; Saleh and Shawky Moiety, 2014; Ege *et al.*, 2019; Aslan *et al.*, 2022; Fujii and Oguchi, 2023). Almost all studies directly tackling the subject of uterine malformations were of poor quality (6/8), with the exception of two studies that utilized 3D ultrasound and accounted for other causes of hormonal disturbances, including hyperandrogenism (Leonhardt *et al.*, 2012; Tokhunts *et al.*, 2022). The detailed quality assessment is available in Supplementary Data File S2.

Due to very high heterogeneity of included studies, we further downgraded the quality of the evidence based on observational studies to very low quality.

## Discussion

This is the first review to summarize the available knowledge on uterine anomalies and morphology in the setting of PCOS. We found that the prevalence of congenital uterine malformations is higher in women with PCOS than in unaffected women, and specific uterine morphological and functional changes are potentially generated by and associated with PCOS. The calculated, general count of uterine anomalies (Table 3) in a non-PCOS population was consistent with previously published research on unselected population (Chan *et al.*, 2011b). The available evidence is of very poor quality: various diagnostic techniques, criteria, and classifications with contrary definitions introduce considerable heterogeneity. A possibly increased prevalence of uterine malformations in women with PCOS may indicate the need for further surveillance in this group. Simultaneously, our results indicate that the scope of the knowledge regarding these issues must be increased and adequately addressed by taking a systematic approach to accomplish the following:

I. Significantly improve current classification systems to enable the reliable assessment of normal, subtle changes in uterine conditions, and congenital uterine malformations;
II. Distinguish between uterine morphological alterations caused by an embryological origin versus those induced by chronic metabolic and hormonal imbalance accompanying PCOS, e.g. hyperandrogenism, and evaluate their association/coexistence;
III. Assess the impact of specific uterine changes on reproductive function and critically evaluate available treatment options; and
IV. Increase the quality of the conducted studies in terms of methodology.

The clinical importance of the reported findings remains inconclusive and requires further debate; however, it highly stimulates the necessity of studies with sound methodology and strict uterine measurements. PCOS is a known cause of subfertility. Furthermore, uterine malformations may affect reproductive outcomes, resulting in a significant increase in recurrent pregnancy loss and preterm births (Saravelos *et al.*, 2008; Chan *et al.*, 2011; Jayaprakasan *et al.*, 2011). This correlation is pronounced especially in women with concomitant infertility (Chan *et al.*, 2011). If the association between PCOS and uterine anomalies was to be confirmed, a targeted ultrasound screening could be suggested in all patients affected.

Currently available studies on uterine anomalies use inadequate assessment methods and classifications, and are prone to a high risk of selection bias in terms of the studied patients and controls. Differences between classification systems and criteria for uterine malformations are poorly addressed (Table 1). A few studies that could not be included in this review link the isolated polycystic ovaries morphology to the occurrence of Müllerian anomalies (MacDougall *et al.*, 1992; Ugur *et al.*, 1995; Appelman *et al.*, 2003). The increased prevalence of congenital uterine malformations was found statistically significant in all but one of the studies (MacDougall *et al.*, 1992), and included scope of arcuate, septate, and bicornuate uteri (Ugur *et al.*, 1995; Appelman *et al.*, 2003).

The authors of the reported research rarely address other potential etiologies of typical PCOS features, such as non-classical congenital adrenal hyperplasia due to 21-hydroxylase deficiency (NCAH). Diseases such as NCAH may share some clinical aspects with PCOS, including polycystic ovarian morphology and ovarian dysfunction. There is a need for more prospective studies with more representative patient groups, preferably selected through simple or stratified random sampling. The controls should include healthy subjects screened for potential additional diagnoses or hormonal treatment.

The androgen-dependent theory of uterine anomalies has been proposed by multiple authors (Panidis *et al.*, 2014; Aslan *et al.*, 2022; Tokhunts *et al.*, 2022; Fujii and Oguchi, 2023). However, only a few articles have adequately addressed the issue of prolonged hyperandrogenism (Tokhunts *et al.*, 2022) and differences between PCOS phenotypes in their methodology (Panidis *et al.*, 2014; Fujii and Oguchi, 2023)—only 65–75% of PCOS patients suffer from elevated androgen levels (Azziz *et al.*, 2004). Other suspected mechanisms leading to altered uterine morphology included androgen exposure *in utero* and elevated AMH levels possibly disrupting Müllerian duct formation during the development stage (Moramezi *et al.*, 2013; Aslan *et al.*, 2020). Pregnant patients with PCOS have statistically higher serum concentrations of AMH, which may disrupt the GnRH receptor signaling and placental metabolism, conditioning the offspring to develop PCOS neuroendocrine and reproductive phenotype later in life (Tata *et al.*, 2018; Piltonen *et al.*, 2019). These changes might be possibly linked to the *Hox* gene, which regulates the differentiation of the Müllerian ducts, and might be modulated by the levels of AMH (Cheng *et al.*, 2011; Aslan *et al.*, 2022). Metabolic disruptions associated with PCOS, such as insulin resistance, diabetes mellitus, and altered uterine perfusion, may also contribute to the potential disturbances of a fetus’s development *in utero* (Schaefer-Graf *et al.*, 2000) as well as influence functional uterine changes later in life (Chekir *et al.*, 2005). All mentioned mechanisms might also be explained by the genetic background of PCOS and its possible inheritance pattern (Kahsar-Miller *et al.*, 2001; Vink *et al.*, 2006; Coviello *et al.*, 2011).

In one study (Tokhunts *et al.*, 2022), some uterine malformations were equally frequent in the PCOS and congenital adrenal hypoplasia groups, as compared with control groups of females with infertility and other endocrine disorders. Another study (Panidis *et al.*, 2014) attributed reduced UV to more severe hyperandrogenemia in the classic PCOS phenotypes; elevated androgen levels decrease uterine blood perfusion and may therefore affect UV. Interestingly, the volume progression connected to age was also seen in the control group (Panidis *et al.*, 2014).

A key limitation of the study is the significant imbalance in infertility rates between groups, indicating an obvious selection bias in original studies. The non-PCOS patients overwhelmingly comprised infertile women (95.7%, 7311/7643) as compared with the PCOS group (56.8%, 1670/2938). Considering that the prevalence of uterine malformations seems to be higher in infertile women than in the general population (8.0% vs 5.5%) (Chan *et al.*, 2011) and that the controls are not representative of unselected non-PCOS women, this bias may lead to underestimating true associations between PCOS and uterine malformations.

Important common limitation refers to the use of inaccurate and unreliable diagnostic tools, which are not currently considered a reference, to assess uterine morphological anomalies. Authors should preferably utilize an adequate imaging method that allows for the visualization of the whole uterus, including the external shape, myometrium thickness, and uterine cavity, while being less invasive and more acceptable to patients; these considerations favor 3-dimensional ultrasonography (Jurkovic *et al.*, 1995; Bermejo *et al.*, 2010; Ludwin *et al.*, 2013, 2015; Saravelos *et al.*, 2017). To blind the outcome advisors, the diagnosis of uterine anomalies should be performed using off-line analysis or pre-acquired 3D datasets. The technique of image acquisition is also important: many of the reported studies assessed only conventional measurements, such as fundal internal indentation depth, angle, and external indentation depth, while omitting recently suggested assessments, such as lateral indentation angle, depth, and T-angle (Salim *et al.*, 2003; Ludwin *et al.*, 2013). The standardization of measurements for diagnosis of uterine anomalies is commonly low or unreported (Ludwin and Martins, 2021). Additionally, different classification systems may vary drastically in their results, and therefore, it is vital that these contrasts should be accounted for during methodology planning and meticulously reported. Existing research based on the oldest and the most popular ASRM/AFS 1988 classification cannot be extrapolated due to the subjective nature of the criteria and the need for more strictly defined measurements. Many common uterine morphologies fail to qualify for any of the arbitrary categories presented by the ASRM/MAC 2021, ESHRE/ESGE, or ASRM 2016 definitions (Ludwin *et al.*, 2015, 2018, 2019, 2020). Moreover, the suggested subclasses of T-shaped uterus (I-shaped and Y-shaped uteri) have no reliable definitions and remain arbitrary. These morphologies are classified as normal/arcuate uterus by the ASRM/AFS 1988, ASRM/MAC 2021, and normal or septate uterus by ESHRE/ESGE.

Some authors suggest surgical reconstruction for the correction of uterine malformations, particularly septate uterus (National Institute for Health and Care Excellence [NICE], 2015a,b; Pfeifer *et al.*, 2016; ESHRE Guideline Group on RPL *et al.*, 2018, 2023; Ludwin and Pfeifer, 2019). However, the benefits associated with the early treatment of these changes have not been fully established (Rikken *et al.*, 2017, 2020a,b; Ludwin, 2020; Saridogan *et al.*, 2020). The available surgical studies on the effectiveness and safety of metroplasty of septate and dysmorphic uteri are not directly related to patients with PCOS. The best-quality studies available include one small RCT and a large cohort study (Rikken *et al.*, 2020b, 2021), both of which showed no benefits of hysteroscopic metroplasty of septate uterus. However, many lower-quality studies reported a dramatically increasing rate of term birth after septum resection (Homer *et al.*, 2000). Studies on metroplasty of septate and dysmorphic uterus are usually critically biased; they consist of before and after observations without the use of a control group (Homer *et al.*, 2000; Coelho Neto *et al.*, 2021; Vercellini *et al.*, 2021). More advanced studies and definitive answers regarding this topic could facilitate access to invasive therapy and decrease the psychological and physiological impact on women affected.

Studies of the uterus exposed to prolonged hormonal imbalance bear a promise of advances in matching the function of the uterus with its ultrasound imaging characteristics. Further research may uncover new details regarding uterine development and facilitate the creation of definitions of a norm and outliers of the uterine structure. Investigating the underlying mechanisms and factors contributing to infertility in these and similar conditions could provide valuable insights into the pathophysiology and potential treatment options for individuals affected by these disorders. The genesis of minor uterine anomalies requires additional attention. If we could confirm that observed changes are, in fact, acquired and modulated by hormonal imbalance/PCOS, future research could explore the possibility of pharmacological treatment. In such case, the restoration of normal uterine morphology could exclude any surgical interventions (Leone and Cammarata, 2021; Ludwin *et al.*, 2021). Additional studies in these areas could potentially transform the entire perception of uterine malformations. However, due to the low quality of available evidence, the results of our work cannot yet be directly translated into the clinical setting.

Research based on uterine morphology might also prove to be beneficial for discovering the pathology and etiology of PCOS as well as the possible influence of hormonal imbalance, e.g. hyperandrogenism on the fetus *in utero*. The repeated reports of an increased prevalence of uterine defects and anomalies seem consistent with the theory of the multi-gene background of this disease.

## Conclusions

Although the available literature suggests an increase in some uterine anomalies in PCOS women, one should not draw meaningful conclusions regarding whether PCOS is associated with uterine size and/or anomalies, since the evidence is still of very low quality. The main existing classification systems constitute a significant limitation, as none allows for reliable and widely accepted diagnosis, particularly for minor uterine malformations. Moreover, there is no scientific foundation for recommending any intervention for women with PCOS based on the observed difference.

## Supplementary Material

deaf117_Supplementary_Data_File_S1

deaf117_Supplementary_Data_File_S2

deaf117_Supplementary_Data_File_S3

deaf117_Supplementary_Data_File_S4

## Data Availability

*The data underlying this article will be shared on reasonable request to the corresponding author.*

## References

[deaf117-B1] Aas‐Eng MK , Langebrekke A, Hudelist G. Complications in operative hysteroscopy—is prevention possible? Acta Obstet Gynecol Scand 2017;96:1399–1403. 28832907 10.1111/aogs.13209

[deaf117-B2] Albdairi AAH , Al-Shalah MAN. Cross-sectional study of the association between polycystic ovary syndrome and uterine septum anomalies. Int J Pharm Res 2021;13:114–118. doi:10.31838/ijpr/2021.13.01.535.

[deaf117-B3] Appelman Z , Hazan Y, Hagay Z. High prevalence of Müllerian anomalies diagnosed by ultrasound in women with polycystic ovaries. J Reprod Med 2003;48:362–364. 12815910

[deaf117-B4] Aslan K , Albayrak O, Bilgic KO, Kasapoglu I, Avcı B, Uncu G. AMH levels may predict for Mullerian anomalies and pregnancy outcomes patients with PCOS. Fertil Steril 2020;114:e407.

[deaf117-B5] Aslan K , Albayrak O, Orhaner A, Kasapoglu I, Uncu G. Incidence of congenital uterine abnormalities in polycystic ovarian syndrome (CONUTA Study). Eur J Obstet Gynecol Reprod Biol 2022;271:183–188. 35219169 10.1016/j.ejogrb.2022.02.012

[deaf117-B6] Azziz R , Woods KS, Reyna R, Key TJ, Knochenhauer ES, Yildiz BO. The prevalence and features of the polycystic ovary syndrome in an unselected population. J Clin Endocrinol Metab 2004;89:2745–2749. 15181052 10.1210/jc.2003-032046

[deaf117-B7] Bermejo C , Martínez Ten P, Cantarero R, Diaz D, Pérez Pedregosa J, Barrón E, Labrador E, Ruiz López L. Three-dimensional ultrasound in the diagnosis of Müllerian duct anomalies and concordance with magnetic resonance imaging. Ultrasound Obstet Gynecol 2010;35:593–601. 20052665 10.1002/uog.7551

[deaf117-B8] Bozdag G , Mumusoglu S, Zengin D, Karabulut E, Yildiz B. The prevalence and phenotypic features of polycystic ovary syndrome: a systematic review and meta-analysis. Hum Reprod 2016;31:2841–2855. 27664216 10.1093/humrep/dew218

[deaf117-B9] Chan YY , Jayaprakasan K, Tan A, Thornton JG, Coomarasamy A, Raine-Fenning NJ. Reproductive outcomes in women with congenital uterine anomalies: a systematic review. Ultrasound Obstet Gynecol 2011a;38:371–382. 21830244 10.1002/uog.10056

[deaf117-B10] Chan YY , Jayaprakasan K, Zamora J, Thornton JG, Raine-Fenning N, Coomarasamy A. The prevalence of congenital uterine anomalies in unselected and high-risk populations: a systematic review. Hum Reprod Update 2011b;17:761–771. 21705770 10.1093/humupd/dmr028 PMC3191936

[deaf117-B11] Chapron C , Fauconnier A, Goffinet F, Bréart G, Dubuisson JB. Laparoscopic surgery is not inherently dangerous for patients presenting with benign gynaecologic pathology. Results of a meta-analysis. Hum Reprod 2002;17:1334–1342. 11980761 10.1093/humrep/17.5.1334

[deaf117-B12] Chekir C , Nakatsuka M, Kamada Y, Noguchi S, Sasaki A, Hiramatsu Y. Impaired uterine perfusion associated with metabolic disorders in women with polycystic ovary syndrome. Acta Obstet Gynecol Scand 2005;84:189–195. 15683382 10.1111/j.0001-6349.2005.00678.x

[deaf117-B13] Cheng Z , Zhu Y, Su D, Wang J, Cheng L, Chen B, Wei Z, Zhou P, Wang B, Ma X et al A novel mutation of HOXA10 in a Chinese woman with a Müllerian duct anomaly. Hum Reprod 2011;26:3197–3201. 21900391 10.1093/humrep/der290

[deaf117-B14] Coelho Neto MA , Ludwin A, Petraglia F, Martins WP. Definition, prevalence, clinical relevance and treatment of T‐shaped uterus: systematic review. Ultrasound Obstet Gynecol 2021;57:366–377. 32898287 10.1002/uog.23108

[deaf117-B15] Coviello AD , Zhuang WV, Lunetta KL, Bhasin S, Ulloor J, Zhang A, Karasik D, Kiel DP, Vasan RS, Murabito JM. Circulating testosterone and SHBG concentrations are heritable in women: the Framingham Heart Study. J Clin Endocrinol Metab 2011;96:E1491–E1495. 21752884 10.1210/jc.2011-0050 PMC3167671

[deaf117-B16] de Zegher F , Francois I, Boehmer AL, Saggese G, Müller J, Hiort O, Sultan C, Clayton P, Brauner R, Cacciari E et al Androgens and fetal growth. Horm Res 1998;50:243–244. 9838248 10.1159/000023284

[deaf117-B17] Ege S , Peker N, Bademkıran MH. The prevalence of uterine anomalies in infertile patients with polycystic ovary syndrome: a retrospective study in a tertiary center in Southeastern Turkey. Turk J Obstet Gynecol 2019;16:224–227. 32231852 10.4274/tjod.galenos.2019.62589 PMC7090257

[deaf117-B18] ESHRE Guideline Group on RPL; Bender Atik R, Christiansen OB, Elson J, Kolte AM, Lewis S, Middeldorp S, Mcheik S, Peramo B, Quenby S et al ESHRE guideline: recurrent pregnancy loss: an update in 2022. Hum Reprod Open 2023;2023:hoad002. 36873081 10.1093/hropen/hoad002 PMC9982362

[deaf117-B19] ESHRE Guideline Group on RPL; Bender Atik R, Christiansen OB, Elson J, Kolte AM, Lewis S, Middeldorp S, Nelen W, Peramo B, Quenby S et al ESHRE guideline: recurrent pregnancy loss. Hum Reprod Open 2018;2018:hoy004. 31486805 10.1093/hropen/hoy004 PMC6276652

[deaf117-B20] Filippou P , Homburg R. Is foetal hyperexposure to androgens a cause of PCOS? Hum Reprod Update 2017;23:421–432. 28531286 10.1093/humupd/dmx013

[deaf117-B21] Fujii S , Oguchi T. Shapes of the uterine cavity are different in women with polycystic ovary syndrome. Reprod Med Biol 2023;22:e12508. 36845000 10.1002/rmb2.12508 PMC9949362

[deaf117-B22] Grimbizis GF , Di Spiezio Sardo A, Saravelos SH, Gordts S, Exacoustos C, Van Schoubroeck D, Bermejo C, Amso NN, Nargund G, Timmerman D et al The Thessaloniki ESHRE/ESGE consensus on diagnosis of female genital anomalies. Hum Reprod 2016;31:2–7. 26537921 10.1093/humrep/dev264

[deaf117-B23] Grimbizis GF , Gordts S, Di Spiezio Sardo A, Brucker S, De Angelis C, Gergolet M, Li T-C, Tanos V, Brolmann H, Gianaroli L et al The ESHRE/ESGE consensus on the classification of female genital tract congenital anomalies. Hum Reprod 2013;28:2032–2044. 23771171 10.1093/humrep/det098 PMC3712660

[deaf117-B24] Homer HA , Li T-C, Cooke ID. The septate uterus: a review of management and reproductive outcome. Fertil Steril 2000;73:1–14. 10632403 10.1016/s0015-0282(99)00480-x

[deaf117-B25] Jayaprakasan K , Chan YY, Sur S, Deb S, Clewes JS, Raine-Fenning NJ. Prevalence of uterine anomalies and their impact on early pregnancy in women conceiving after assisted reproduction treatment. Ultrasound Obstet Gynecol 2011;37:727–732. 21337662 10.1002/uog.8968

[deaf117-B26] Jurkovic D , Geipel A, Gruboeck K, Jauniaux E, Natucci M, Campbell S. Three-dimensional ultrasound for the assessment of uterine anatomy and detection of congenital anomalies: a comparison with hysterosalpingography and two-dimensional sonography. Ultrasound Obstet Gynecol 1995;5:233–237. 7600203 10.1046/j.1469-0705.1995.05040233.x

[deaf117-B27] Kahsar-Miller MD , Nixon C, Boots LR, Go RC, Azziz R. Prevalence of polycystic ovary syndrome (PCOS) in first-degree relatives of patients with PCOS. Fertil Steril 2001;75:53–58. 11163816 10.1016/s0015-0282(00)01662-9

[deaf117-B28] Kaufman RH , Binder GL, Gray PM, Adam E. Upper genital tract changes associated with exposure in utero to diethylstilbestrol. Am J Obstet Gynecol 1977;128:51–59. 851159 10.1016/0002-9378(77)90294-0

[deaf117-B29] Kawano M , Fuke Y, Nakayama T. [Ultrasonic findings in polycystic ovary]. Nihon Sanka Fujinka Gakkai Zasshi 1987;39:56–62. 3546532

[deaf117-B30] Knez J , Saridogan E, Van Den Bosch T, Mavrelos D, Ambler G, Jurkovic D. ESHRE/ESGE female genital tract anomalies classification system—the potential impact of discarding arcuate uterus on clinical practice. Hum Reprod 2018;33:600–606. 29514262 10.1093/humrep/dey043

[deaf117-B31] Leone FPG , Cammarata S. Oral contraception and overdiagnosis of T‐shaped uterus: keep calm and rescan. Ultrasound Obstet Gynecol 2021;57:655–656. 33793000 10.1002/uog.23626

[deaf117-B32] Leonhardt H , Gull B, Kishimoto K, Kataoka M, Nilsson L, Janson PO, Stener-Victorin E, Hellström M. Uterine morphology and peristalsis in women with polycystic ovary syndrome. Acta Radiol 2012;53:1195–1201. 23081959 10.1258/ar.2012.120384

[deaf117-B33] Ludwin A. Septum resection does not improve reproductive outcomes: truly? Hum Reprod 2020;35:1495–1498. 32568394 10.1093/humrep/deaa142

[deaf117-B34] Ludwin A , Coelho Neto MA, Ludwin I, Nastri CO, Costa W, Acién M, Alcazar JL, Benacerraf B, Condous G, DeCherney A et al Congenital Uterine Malformation by Experts (CUME): diagnostic criteria for T‐shaped uterus. Ultrasound Obstet Gynecol 2020;55:815–829. 31432589 10.1002/uog.20845

[deaf117-B35] Ludwin A , Ludwin I. Comparison of the ESHRE-ESGE and ASRM classifications of Mullerian duct anomalies in everyday practice. Hum Reprod 2015;30:569–580. 25534461 10.1093/humrep/deu344 PMC4325671

[deaf117-B36] Ludwin A , Ludwin I. Reliability of hysteroscopy-based diagnosis of septate, arcuate and normal uterus: estimate or guestimate? Hum Reprod 2016;31:1376–1377. 27103072 10.1093/humrep/dew086

[deaf117-B37] Ludwin A , Ludwin I, Coelho Neto MA, Nastri CO, Bhagavath B, Lindheim SR, Martins WP. Septate uterus according to ESHRE/ESGE, ASRM and CUME definitions: association with infertility and miscarriage, cost and warnings for women and healthcare systems. Ultrasound Obstet Gynecol 2019;54:800–814. 30977223 10.1002/uog.20291

[deaf117-B38] Ludwin A , Ludwin I, Kudla M, Kottner J. Reliability of the European Society of Human Reproduction and Embryology/European Society for Gynaecological Endoscopy and American Society for Reproductive Medicine classification systems for congenital uterine anomalies detected using three-dimensional ultrasonography. Fertil Steril 2015;104:688–697.e8. 26158905 10.1016/j.fertnstert.2015.06.019

[deaf117-B39] Ludwin A , Ludwin I, Pitynski K, Jach R, Banas T. Are the ESHRE/ESGE criteria of female genital anomalies for diagnosis of septate uterus appropriate? Hum Reprod 2014;29:867–868. 24480714 10.1093/humrep/deu001

[deaf117-B40] Ludwin A , Martins WP. Correct measurement of uterine fundal internal indentation depth and angle: an important but overlooked issue for precise diagnosis of uterine anomalies. Ultrasound Obstet Gynecol 2021;58:497–499. 32851686 10.1002/uog.22192

[deaf117-B41] Ludwin A , Martins WP, Nastri CO, Ludwin I, Coelho Neto MA, Leitão VM, Acién M, Alcazar JL, Benacerraf B, Condous G et al Congenital Uterine Malformation by Experts (CUME): better criteria for distinguishing between normal/arcuate and septate uterus? Ultrasound Obstet Gynecol 2018;51:101–109. 29024135 10.1002/uog.18923

[deaf117-B42] Ludwin A , Neto MC, Martins WP. Reply: T‐shaped uterus after oral contraception—considering myometrial contractions, endometrial volume and 3D saline contrast sonohysterography in diagnosis. Ultrasound Obstet Gynecol 2021;57:656–658. 33792999 10.1002/uog.23627

[deaf117-B43] Ludwin A , Pfeifer SM. Reproductive surgery for Müllerian anomalies: a review of progress in the last decade. Fertil Steril 2019;112:408–416. 31446900 10.1016/j.fertnstert.2019.07.005

[deaf117-B44] Ludwin A , Pityński K, Ludwin I, Banas T, Knafel A. Two- and three-dimensional ultrasonography and sonohysterography versus hysteroscopy with laparoscopy in the differential diagnosis of septate, bicornuate, and arcuate uteri. J Minim Invasive Gynecol 2013;20:90–99. 23312248 10.1016/j.jmig.2012.09.011

[deaf117-B45] Macdougall MJ , Patel A, Jacobs HS. Polycystic ovaries in association with Müllerian duct anomalies. BJOG 1992;99:520–521. 10.1111/j.1471-0528.1992.tb13796.x 1637773

[deaf117-B46] Mata DA , Ramos MA, Bansal N, Khan R, Guille C, Di Angelantonio E, Sen S. Prevalence of depression and depressive symptoms among resident physicians. JAMA 2015;314:2373–2383. 26647259 10.1001/jama.2015.15845 PMC4866499

[deaf117-B47] Moramezi F , Barati M, Shahbazian N, Golbabaei M, Hemadi M. Sonographic evaluation of Mullerian anomalies in women with polycystic ovaries. Health N Hav 2013;05:1313–1317.

[deaf117-B48] National Institute for Health and Care Excellence [NICE]. Hysteroscopic metroplasty of a uterine septum for recurrent miscarriage. *NICE Guidelines*. Manchester, UK: NICE, 2015a. https://www.nice.org.uk/guidance/ipg510 (5 February 2025, date last accessed).

[deaf117-B49] National Institute for Health and Care Excellence [NICE]. Hysteroscopic metroplasty of a uterine septum for primary infertility. *NICE Guideline*. Manchester, UK: NICE, 2015b. https://www.nice.org.uk/guidance/ipg509 (5 February 2025, date last accessed).

[deaf117-B50] Orsini LF , Venturoli S, Lorusso R, Pluchinotta V, Paradisi R, Bovicelli L. Ultrasonic findings in polycystic ovarian disease. Fertil Steril 1985;43:709–714. 3888678 10.1016/s0015-0282(16)48552-3

[deaf117-B51] Page MJ , McKenzie JE, Bossuyt PM, Boutron I, Hoffmann TC, Mulrow CD, Shamseer L, Tetzlaff JM, Akl EA, Brennan SE et al The PRISMA 2020 statement: an updated guideline for reporting systematic reviews. BMJ 2021;372:n71. 33782057 10.1136/bmj.n71 PMC8005924

[deaf117-B52] Panidis D , Tziomalos K, Papadakis E, Vosnakis C, Betsas G, Tsourdi E, Katsikis I. Uterine volume and endometrial thickness in the early follicular phase in patients with polycystic ovary syndrome. Endocr Pract 2014;20:540–547. 24325993 10.4158/EP13058.OR

[deaf117-B53] Pfeifer S , Butts S, Dumesic D, Gracia C, Vernon M, Fossum G, La Barbera A, Mersereau J, Odem R, Penzias A et al Uterine septum: a guideline. Fertil Steril 2016;106:530–540. 27235766 10.1016/j.fertnstert.2016.05.014

[deaf117-B54] Pfeifer SM , Attaran M, Goldstein J, Lindheim SR, Petrozza JC, Rackow BW, Siegelman E, Troiano R, Winter T, Zuckerman A et al ASRM Müllerian anomalies classification 2021. Fertil Steril 2021;116:1238–1252. 34756327 10.1016/j.fertnstert.2021.09.025

[deaf117-B55] Piltonen TT , Giacobini P, Edvinsson Å, Hustad S, Lager S, Morin-Papunen L, Tapanainen JS, Sundström-Poromaa I, Arffman RK. Circulating antiMüllerian hormone and steroid hormone levels remain high in pregnant women with polycystic ovary syndrome at term. Fertil Steril 2019;111:588–596.e1. 30630591 10.1016/j.fertnstert.2018.11.028

[deaf117-B56] Rikken JF , Kowalik CR, Emanuel MH, Mol BWJ, van der Veen F, van Wely M, Goddijn M. Septum resection for women of reproductive age with a septate uterus. Cochrane Database Syst Rev 2017;1:CD008576. 28093720 10.1002/14651858.CD008576.pub4 PMC6464821

[deaf117-B57] Rikken JFW , Kowalik CR, Emanuel MH, Bongers MY, Spinder T, Jansen FW, Mulders AGMGJ, Padmehr R, Clark TJ, van Vliet HA et al Septum resection versus expectant management in women with a septate uterus: an international multicentre open-label randomized controlled trial. Hum Reprod 2021;36:1260–1267. 33793794 10.1093/humrep/deab037 PMC8058590

[deaf117-B58] Rikken JFW , van der Veen F, van Wely M, Goddijn M. Reply: septum resection, seriously? Hum Reprod 2020a;35:2630–2631. 32974641 10.1093/humrep/deaa231

[deaf117-B59] Rikken JFW , Verhorstert KWJ, Emanuel MH, Bongers MY, Spinder T, Kuchenbecker W, Jansen FW, van der Steeg JW, Janssen CAH, Kapiteijn K et al Septum resection in women with a septate uterus: a cohort study. Hum Reprod 2020b;35:1578–1588. 32353142 10.1093/humrep/dez284 PMC7368397

[deaf117-B60] Risal S , Pei Y, Lu H, Manti M, Fornes R, Pui H-P, Zhao Z, Massart J, Ohlsson C, Lindgren E et al Prenatal androgen exposure and transgenerational susceptibility to polycystic ovary syndrome. Nat Med 2019;25:1894–1904. 31792459 10.1038/s41591-019-0666-1

[deaf117-B61] Roly ZY , Backhouse B, Cutting A, Tan TY, Sinclair AH, Ayers KL, Major AT, Smith CA. The cell biology and molecular genetics of Müllerian duct development. Wiley Interdiscip Rev Dev Biol 2018;7:e310. 29350886 10.1002/wdev.310

[deaf117-B62] Rotterdam ESHRE/ASRM-Sponsored PCOS Consensus Workshop Group. Revised 2003 consensus on diagnostic criteria and long-term health risks related to polycystic ovary syndrome. Fertil Steril 2004;81:19–25. 10.1016/j.fertnstert.2003.10.004 14711538

[deaf117-B63] Saleh HA , Shawky Moiety FM. Polycystic ovarian syndrome and congenital uterine anomalies: the hidden common player. Arch Gynecol Obstet 2014;290:355–360. 24615567 10.1007/s00404-014-3193-9

[deaf117-B64] Salim R , Woelfer B, Backos M, Regan L, Jurkovic D. Reproducibility of three-dimensional ultrasound diagnosis of congenital uterine anomalies. Ultrasound Obstet Gynecol 2003;21:578–582. 12808675 10.1002/uog.127

[deaf117-B65] Saravelos SH , Cocksedge KA, Li T-C. Prevalence and diagnosis of congenital uterine anomalies in women with reproductive failure: a critical appraisal. Hum Reprod Update 2008;14:415–429. 18539641 10.1093/humupd/dmn018

[deaf117-B66] Saravelos SH , Jayaprakasan K, Ojha K, Li T-C. Assessment of the uterus with three-dimensional ultrasound in women undergoing ART. Hum Reprod Update 2017;23:188–210. 28007752 10.1093/humupd/dmw040

[deaf117-B67] Saridogan E , Mavrelos D, Jurkovic D. To decide on the value of hysteroscopic septum resection we need prospective data. Hum Reprod 2020;35:2627–2627. 10.1093/humrep/deaa229 32968759

[deaf117-B68] Schaefer-Graf UM , Buchanan TA, Xiang A, Songster G, Montoro M, Kjos SL. Patterns of congenital anomalies and relationship to initial maternal fasting glucose levels in pregnancies complicated by type 2 and gestational diabetes. Am J Obstet Gynecol 2000;182:313–320. 10694330 10.1016/s0002-9378(00)70217-1

[deaf117-B69] Smit JG , Kasius JC, Eijkemans MJC, Veersema S, Fatemi HM, Santbrink van EJP, Campo R, Broekmans FJM. The international agreement study on the diagnosis of the septate uterus at office hysteroscopy in infertile patients. Fertil Steril 2013;99:2108–2113.e2. 23499151 10.1016/j.fertnstert.2013.02.027

[deaf117-B70] Smit JG , Overdijkink S, Mol BW, Kasius JC, Torrance HL, Eijkemans MJC, Bongers M, Emanuel MH, Vleugels M, Broekmans FJM. The impact of diagnostic criteria on the reproducibility of the hysteroscopic diagnosis of the septate uterus: a randomized controlled trial. Hum Reprod 2015;30:1323–1330. 25904634 10.1093/humrep/dev082

[deaf117-B71] Smit JG , Torrance HL, Eijkemans MJC, Broekmans FJM. Reply: reliability of hysteroscopy-based diagnosis of septate, arcuate and normal uterus: estimate or guestimate? Hum Reprod 2016;31:1377–1378. 27103073 10.1093/humrep/dew087

[deaf117-B72] Stang A. Critical evaluation of the Newcastle-Ottawa scale for the assessment of the quality of nonrandomized studies in meta-analyses. Eur J Epidemiol 2010;25:603–605. 20652370 10.1007/s10654-010-9491-z

[deaf117-B73] Tata B , Mimouni NEH, Barbotin A-L, Malone SA, Loyens A, Pigny P, Dewailly D, Catteau-Jonard S, Sundström-Poromaa I, Piltonen TT et al Elevated prenatal anti-Müllerian hormone reprograms the fetus and induces polycystic ovary syndrome in adulthood. Nat Med 2018;24:834–846. 29760445 10.1038/s41591-018-0035-5 PMC6098696

[deaf117-B74] The American Fertility Society classifications of adnexal adhesions, distal tubal occlusion, tubal occlusion secondary to tubal ligation, tubal pregnancies, Müllerian anomalies and intrauterine adhesions. Fertil Steril 1988;49:944–955. 3371491 10.1016/s0015-0282(16)59942-7

[deaf117-B75] Tokhunts K , Adamyan M, Chopikyan A, Kayfajyan K, Khudaverdyan A, Tumanyan A. Is I-shaped uterus more common in patients with hyperandrogenism? Eur J Obstet Gynecol Reprod Biol 2022;272:116–122. 35303673 10.1016/j.ejogrb.2022.03.018

[deaf117-B76] Traiman P , Saldiva P, Haiashi A, Franco M. Criteria for the diagnosis of diffuse uterine myohypertrophy. Int J Gynaecol Obstet 1996;54:31–36. 8842815 10.1016/0020-7292(96)02676-8

[deaf117-B77] Ugur M , Karakaya S, Zorlu G, Arslan S, Gülerman C, Kükner S, Gökmen O. Polycystic ovaries in association with Müllerian anomalies. Eur J Obstet Gynecol Reprod Biol 1995;62:57–59. 7493710 10.1016/0301-2115(95)02157-3

[deaf117-B78] Vercellini P , Chiaffarino F, Parazzini F. ‘It’s all too much’†: the shadow of overtreatment looms over hysteroscopic metroplasty for septate uterus. Hum Reprod 2021;36:1166–1170. 33793818 10.1093/humrep/deab081

[deaf117-B79] Vink JM , Sadrzadeh S, Lambalk CB, Boomsma DI. Heritability of polycystic ovary syndrome in a Dutch twin-family study. J Clin Endocrinol Metab 2006;91:2100–2104. 16219714 10.1210/jc.2005-1494

[deaf117-B80] Youm HS , Choi YS, Han HD. In vitro fertilization and embryo transfer outcomes in relation to myometrial thickness. J Assist Reprod Genet 2011;28:1135–1140. 21947758 10.1007/s10815-011-9640-7 PMC3224176

