# Supplementary material for: Uterine morphology and anomalies in women with and without polycystic ovary syndrome: a systematic review and meta-analysis
Source: Hum Reprod. 2025 Jun 19;40(9):1629–42. doi: 10.1093/humrep/deaf117 (PMC12408902; doi:10.1093/humrep/deaf117)
Supplement: deaf117_Supplementary_Data_File_S1 [file deaf117_supplementary_data_file_s1.docx]

**Supplementary Data File S1.** Search strategy.

**Polycystic ovary syndrome**

1. Hyperandrogenism
2. PCOS
3. Polycystic ovary syndrome
4. Stein Leventhal syndrome
5. Functional ovarian hyperandrogenism
6. Ovarian hyperthecosis
7. Sclerocystic ovary syndrome
8. Polycystic ovary disease
9. OR / 1 – 8

**Morphology**

1. Morphology
2. Congenital anomalies
3. Congenital malformation*
4. Measurement*
5. Dimension*
6. Shape
7. Volume
8. Anomalies
9. Mullerian anomalies
10. OR / 10 – 18

**Uterus**

1. Uterus
2. Uteri
3. Uterine
4. OR / 20-22

**Combined search**

1. #9 AND #19 AND #23

(("hyperandrogenism"[All Fields]) OR ("pcos"[All Fields]) OR ("polycystic ovary syndrome"[All Fields]) OR ("stein leventhal syndrome"[All Fields]) OR ("functional ovarian hyperandrogenism"[All Fields]) OR ("ovarian hyperthecosis"[All Fields]) OR ("sclerocystic ovary syndrome"[All Fields]) OR ("polycystic ovary disease"[All Fields])) AND (("morphology"[All Fields]) OR ("congenital anomalies"[All Fields]) OR ("congenital malformations"[All Fields]) OR ("measurement*"[All Fields]) OR ("dimension*"[All Fields]) OR ("volume"[All Fields]) OR ("anomalies"[All Fields]) OR ("mullerian anomalies"[All Fields]) OR ("shape"[All Fields])) AND (("uterus"[All Fields]) OR ("uterine"[All Fields]) OR ("uteri"[All Fields]))
