# Supplementary material for: Uterine morphology and anomalies in women with and without polycystic ovary syndrome: a systematic review and meta-analysis
Source: Hum Reprod. 2025 Jun 19;40(9):1629–42. doi: 10.1093/humrep/deaf117 (PMC12408902; doi:10.1093/humrep/deaf117)
Supplement: deaf117_Supplementary_Data_File_S2 [file deaf117_supplementary_data_file_s2.doc]

**Supplementary Data File S2.** A Modified Newcastle-Ottawa Quality assessment scale

**A MODIFIED NEWCASTLE - OTTAWA QUALITY ASSESSMENT SCALE**

Note: A study can be awarded a maximum of one star for each numbered item.

**Selection**

1) Is the case definition adequate?

a) yes, with independent validation (e.g. uses adequate criteria to assess the presence of PCOS) **✸**

b) partially yes, eg record linkage or based on self-reports, does not utilize adequate PCOS criteria

c) no description

2) Representativeness of the cases

Consider the choice of cases and the process of enrolment.

a) consecutive or obviously representative series of cases **✸**

b) potential for selection biases or not stated

3) Selection of Controls

Consider the choice of controls and the process of enrolment.

a) normal controls **✸**

b) hospital, infertility-treated controls

c) no description

4) Selection of Cases

Consider whether authors performed additional tests or relied solely on medical history.

a) authors accounted for other causes of hyperandrogenism or hormonal disorders **✸**

b) no description of source

**Comparability**

1. Comparability of cases and controls on the basis of the design or analysis

Does the study control for similar source of the populations, similar characteristics?

a) study controls for differences within the populations**✸**

b) study does not control for differences within the populations.

**Outcomes**

1. Ascertainment of outcome

Consider whether study uses appropriate method of assessment for given measurement (e.g., 3D TVUS, MRI), who performed the examinations, the time of examinations, the quality of records.

a) secure record, adequate methods**✸**

e) no description, inappropriate methods.

2) Same method of ascertainment for cases and controls

a) yes **✸**

b) no

3) Objective assessment of outcome

a) Utilizes classification systems (if available), or/and describes the rationale used in detail**✸**

b) subjective assessment, no description

**Statistics**

1. Quality of descriptive statistics reporting:
2. Reported descriptive statistics to describe the population (e.g., age) with proper measures of dispersion (e.g., mean, standard deviation). **✸**
3. Descriptive statistics were not reported, were incomplete, or did not include proper measures of dispersion.

| ID | Study name | Discussion result | Source type |
| --- | --- | --- | --- |
| 1 | Aslan et al., 2022 | 6 | Article |
| 2 | Ege et al., 2020 | 3 | Article |
| 3 | [Fujii and Oguchi, 2023](https://doi.org/10.1002/rmb2.12508) | 6 | Article |
| 4 | Saleh et al.., 2014 | 4 | Article |
| 5 | [Tokhunts et al., 2022](https://doi.org/10.1016/j.ejogrb.2022.03.018) | 7 | Article |
| 6 | Moramezi et al., 2013 | 5 | Article |
| 7 | Albdairi et al., 2021 | 4 | Article |
| 8 | Kawano et al., 1987 | 2 | Abstract |
| 9 | [Panidis et al., 2014](https://doi.org/10.4158/EP13058.OR) | 8 | Article |
| 10 | Orsini et al., 1985 | 4 | Article |
| 11 | [Leonhardt et al., 2012](https://doi.org/10.1258/ar.2012.120384) | 8 | Article |
