# Supplementary material for: Uterine morphology and anomalies in women with and without polycystic ovary syndrome: a systematic review and meta-analysis
Source: Hum Reprod. 2025 Jun 19;40(9):1629–42. doi: 10.1093/humrep/deaf117 (PMC12408902; doi:10.1093/humrep/deaf117)
Supplement: deaf117_Supplementary_Data_File_S3 [file deaf117_supplementary_data_file_s3.docx]

**Supplementary Data File S3.** Records sought for retrival

1. Adams J, Reginald PW, Franks S, Wadsworth J, Beard RW. Uterine size and endometrial thickness and the significance of cystic ovaries in women with pelvic pain due to congestion. Br J Obstet Gynaecol. 1990;97(7):583-587. doi:10.1111/j.1471-0528.1990.tb02544.x
2. Al-Rshoud F, Kilani R, Al-Asali F, Alsharaydeh I, Mahfouz IA. The Prevalence of uterine septum in polycystic ovarian syndrome (PCOS), a series of 49 cases. Jordan Medical Journal. 2020;54:3 (131-136)
3. Albdairi AAH, Al-Shalah MAN. Cross‐sectional study of the association between polycystic ovary syndrome and uterine septum anomalies. International Journal of Pharmaceutical Research. 2021;13:1 (3747-3751)
4. Albdairi AAH, Al-Shalah MAN. Study of the association between the congenital uterine septum and polycystic ovarian syndrome in infertility tertiary center in iraq. Archivos Venezolanos de Farmacologia y Terapeutica. 2021 40:4 (384-388)
5. Appelman Z, Hazan Y, Hagay Z. High prevalence of müllerian anomalies diagnosed by ultrasound in women with polycystic ovaries. J Reprod Med. 2003;48(5):362-364.
6. *Aslan K, Albayrak O, Bilgic KO, Kasapoglu I, Avc Bı, Gürkan U. AMH LEVELS MAY PREDICT FOR MULLERIAN ANOMALIES AND PREGNANCY OUTCOMES PATIENTS WITH PCOS. Fertility and sterility. 2020;114(3):e407. doi:https://doi.org/10.1016/j.fertnstert.2020.08.1191*
7. Aslan K, Albayrak O, Orhaner A, Kasapoglu I, Uncu G. Incidence of congenital uterine abnormalities in polycystic ovarian syndrome (CONUTA Study). Eur J Obstet Gynecol Reprod Biol. 2022;271:183-188. doi:10.1016/j.ejogrb.2022.02.012
8. Battaglia C, Genazzani AD, Artini PG, Salvatori M, Giulini S, Volpe A. Ultrasonographic and color Doppler analysis in the treatment of polycystic ovary syndrome. Ultrasound Obstet Gynecol. 1998;12(3):180-187. doi:10.1046/j.1469-0705.1998.12030180.x
9. Battaglia C, Mancini F, Persico N, Zaccaria V, de Aloysio D. Ultrasound evaluation of PCO, PCOS and OHSS. Reprod Biomed Online. 2004;9(6):614-619. doi:10.1016/s1472-6483(10)61770-1
10. ‌Chen H, Zhan WW, Yang ZF, La DD, Chen C. Ultrasonic features and correlation with endocrine profiles in polycystic ovary syndrome patients with or without insulin resistance. Chinese Journal of Medical Imaging Technology. 2014;30:4 (595-599)
11. Dolz M, Osborne NG, Blanes J, et al. Polycystic ovarian syndrome: assessment with color Doppler angiography and three-dimensional ultrasonography. J Ultrasound Med. 1999;18(4):303-313. doi:10.7863/jum.1999.18.4.303
12. Dwivedi AND, Ganesh V, Shukla RC, Jain M, Kumar I. Colour Doppler evaluation of uterine and ovarian blood flow in patients of polycystic ovarian disease and post-treatment changes. Clin Radiol. 2020;75(10):772-779. doi:10.1016/j.crad.2020.05.023
13. Ege S, Peker N, Bademkıran MH. The prevalence of uterine anomalies in infertile patients with polycystic ovary syndrome: A retrospective study in a tertiary center in Southeastern Turkey. Turk J Obstet Gynecol. 2019;16(4):224-227. doi:10.4274/tjod.galenos.2019.62589
14. Fujii S, Oguchi T. Shapes of the uterine cavity are different in women with polycystic ovary syndrome. Reprod Med Biol. 2023;22(1):e12508. Published 2023 Feb 23. doi:10.1002/rmb2.12508
15. González LS, Artibani M, Ahmed AA. Studying Müllerian duct anomalies - from cataloguing phenotypes to discovering causation. Dis Model Mech. 2021;14(6):dmm047977. doi:10.1242/dmm.047977
16. Kalantari SA, Role of Doppler ultrasonography and 3D Ultrasound in Female Infertility. Iranian Journal of Medical Sciences. 2023;48:1 (7) Supplement
17. Kaproth-Joslin K, Dogra V. Imaging of female infertility: a pictorial guide to the hysterosalpingography, ultrasonography, and magnetic resonance imaging findings of the congenital and acquired causes of female infertility. Radiol Clin North Am. 2013;51(6):967-981. doi:10.1016/j.rcl.2013.07.002
18. Kawano M, Fuke Y, Nakayama T. [Ultrasonic findings in polycystic ovary]. Nihon Sanka Fujinka Gakkai Zasshi. 1987;39(1):56-62.
19. Leonhardt H, Gull B, Kishimoto K, Kataoka M, Stener-Victorin E, Hellström M. Uterine peristalsis and morphology in women with polycystic ovary syndrome evaluated by magnetic resonance imaging. Human Reproduction. 2011;26 (i332) SUPPL. 1
20. Leonhardt H, Gull B, Kishimoto K, et al. Uterine morphology and peristalsis in women with polycystic ovary syndrome. Acta Radiol. 2012;53(10):1195-1201. doi:10.1258/ar.2012.120384
21. Naredi N, Sharma R, Gurmeet P. Can Three-Dimensional Transvaginal Sonography Replace Office Hysteroscopy in Detecting Uterine Abnormalities in Infertility Patients?. J Hum Reprod Sci. 2021;14(4):392-399. doi:10.4103/jhrs.jhrs_97_21
22. Orsini LF, Venturoli S, Lorusso R, Pluchinotta V, Paradisi R, Bovicelli L. Ultrasonic findings in polycystic ovarian disease. Fertil Steril. 1985;43(5):709-714. doi:10.1016/s0015-0282(16)48552-3
23. Panidis D, Tziomalos K, Papadakis E, et al. Uterine volume and endometrial thickness in the early follicular phase in patients with polycystic ovary syndrome. Endocr Pract. 2014;20(6):540-547. doi:10.4158/EP13058.OR
24. Raine-Fenning N, Fleischer AC. Clarifying the role of three-dimensional transvaginal sonography in reproductive medicine: an evidenced-based appraisal. J Exp Clin Assist Reprod. 2005;2:10. Published 2005 Aug 11. doi:10.1186/1743-1050-2-10
25. Revel A, Abramov Y, Yagel S, Nadjari M. Utero-ovarian morphology and blood flow after tubal ligation by the Pomeroy technique. Contraception. 2004;69(2):151-156. doi:10.1016/j.contraception.2003.09.016
26. Saleh HA, Shawky Moiety FM. Polycystic ovarian syndrome and congenital uterine anomalies: the hidden common player. Arch Gynecol Obstet. 2014;290(2):355-360. doi:10.1007/s00404-014-3193-9
27. Shah B, Parnell L, Milla S, Kessler M, David R. Endometrial thickness, uterine, and ovarian ultrasonographic features in adolescents with polycystic ovarian syndrome. J Pediatr Adolesc Gynecol. 2010;23(3):146-152. doi:10.1016/j.jpag.2009.07.002
28. Siam S, Soliman BS. Combined laparoscopy and hysteroscopy for the detection of female genital system anomalies: Results of 3,811 infertile women. Journal of Reproductive Medicine. 2014;59:6 (542-546)
29. Sørensen SS. Hysteroscopic evaluation and endocrinological aspects of women with müllerian anomalies and oligomenorrhea. Int J Fertil. 1987;32(6):445-452.
30. Tan SL. Clinical applications of Doppler and three-dimensional ultrasound in assisted reproductive technology. Ultrasound Obstet Gynecol. 1999;13(3):153-156. doi:10.1046/j.1469-0705.1999.13030153.x
31. Tokhunts K, Adamyan M, Chopikyan A, Kayfajyan K, Khudaverdyan A, Tumanyan A. Is I-shaped uterus more common in patients with hyperandrogenism?. Eur J Obstet Gynecol Reprod Biol. 2022;272:116-122. doi:10.1016/j.ejogrb.2022.03.018
32. Tugrul S, Oral O, Guclu M, Kutlu T, Uslu H, Pekin O. Significance of Doppler ultrasonography in the diagnosis of polycystic ovary syndrome. Clinical and Experimental Obstetrics and Gynecology. 2006;33:3 (154-158)
33. Ugur M, Karakaya S, Zorlu G, et al. Polycystic ovaries in association with müllerian anomalies. Eur J Obstet Gynecol Reprod Biol. 1995;62(1):57-59. doi:10.1016/0301-2115(95)02157-3
34. Usmani A, Islam ZU, Akhtar Z. Comparison of female reproductive organs morphology between fertile and infertile women with polycystic ovaries. Journal of Postgraduate Medical Institute. 2013;27:1 (48-54)
35. Usmani A, Rehman R, Qamar A. Effect of age on uterine and ovarian morphology with Polycystic Ovaries. Journal of the Pakistan Medical Association. 2014;64:10 (1119-1122)
36. Venturoli S, Paradisi R, Saviotti E, et al. Ultrasound study of ovarian and uterine morphology in women with polycystic ovary syndrome before, during and after treatment with cyproterone acetate and ethinyloestradiol. Arch Gynecol. 1985;237(1):1-10. doi:10.1007/BF02133945
37. Wang M, Dai Q. Application of three-dimensional ultrasound in assisted reproductive medicine. Chinese Journal of Medical Imaging Technology; 2011. Volume 27, Issue 9, Pages 1922 – 1925
38. Yang M, Zhang F, Wu K, et al. Müllerian Duct Anomalies and Anti-Müllerian Hormone Levels in Women With Polycystic Ovary Syndrome. Cureus. 2023;15(8):e43848. Published 2023 Aug 21. doi:10.7759/cureus.43848
39. Younesi L, Safarpour Lima Z, Akbari Sene A, Hosseini Jebelli Z, Amjad G. Comparison of uterine and ovarian stromal blood flow in patients with polycystic ovarian syndrome. Endocr Connect. 2019;8(1):50-56. doi:10.1530/EC-18-0423
