# Supplementary material for: Uterine morphology and anomalies in women with and without polycystic ovary syndrome: a systematic review and meta-analysis
Source: Hum Reprod. 2025 Jun 19;40(9):1629–42. doi: 10.1093/humrep/deaf117 (PMC12408902; doi:10.1093/humrep/deaf117)
Supplement: deaf117_Supplementary_Data_File_S4 [file deaf117_supplementary_data_file_s4.docx]

**Supplementary Data File S4**. Metaanalysis details.

1. All uterine anomalies


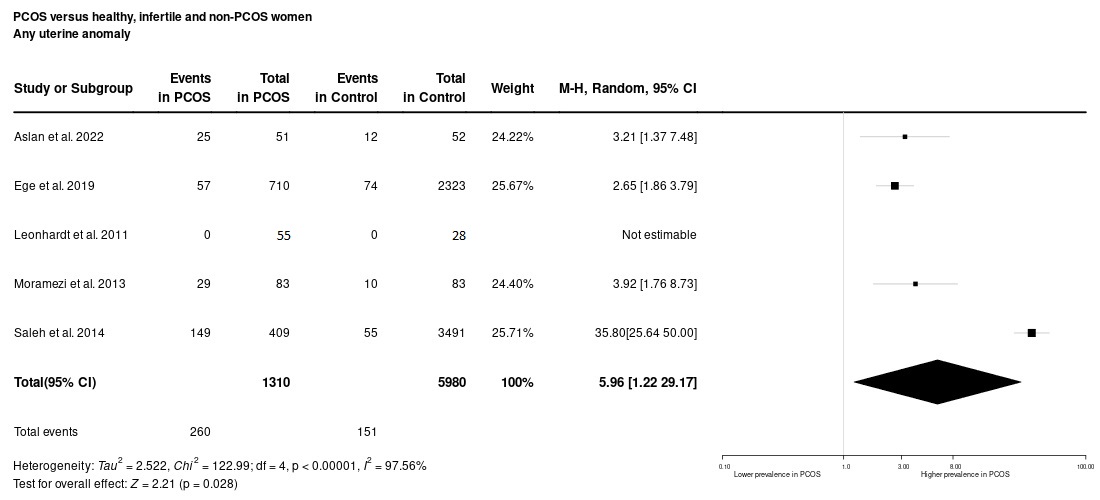


1.
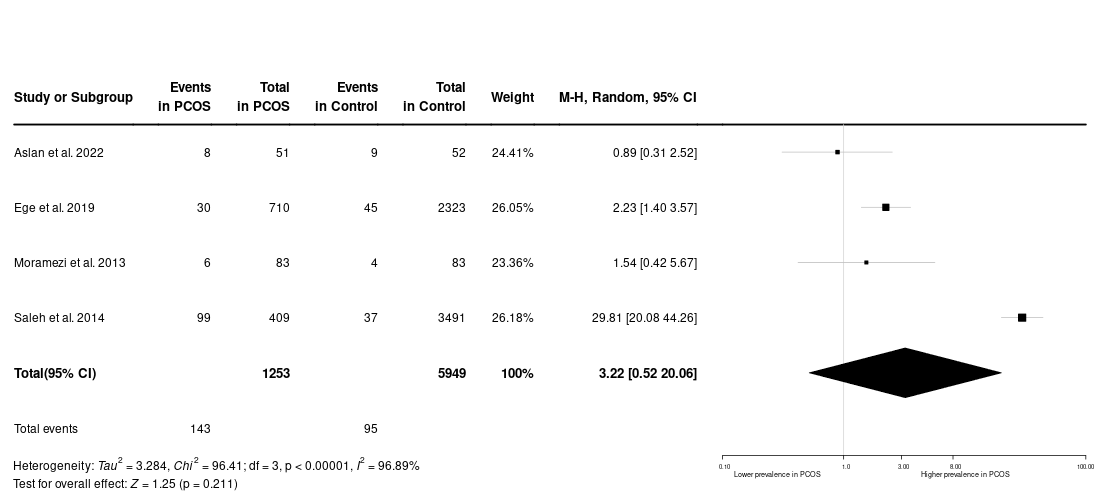
Arcuate uterus
2. Septate uterus


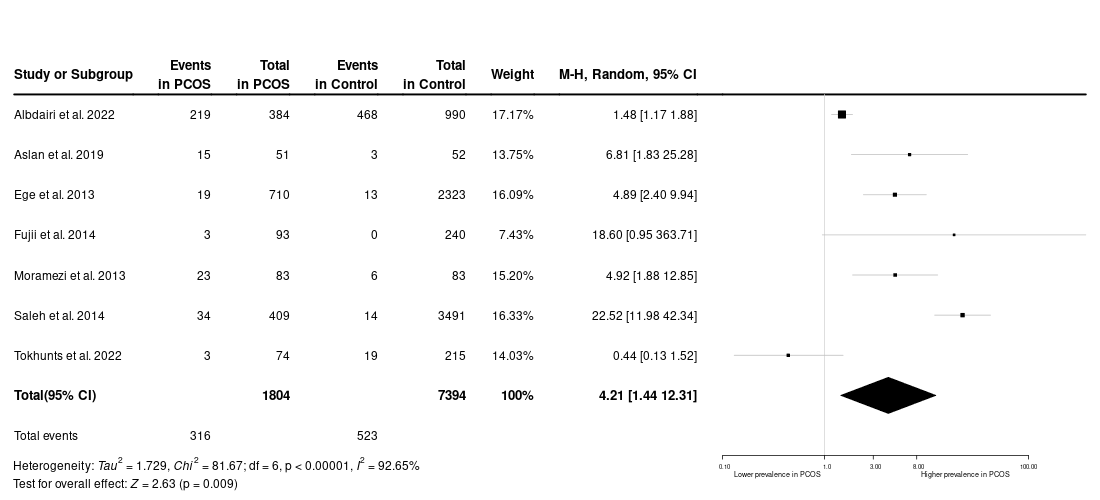


1.
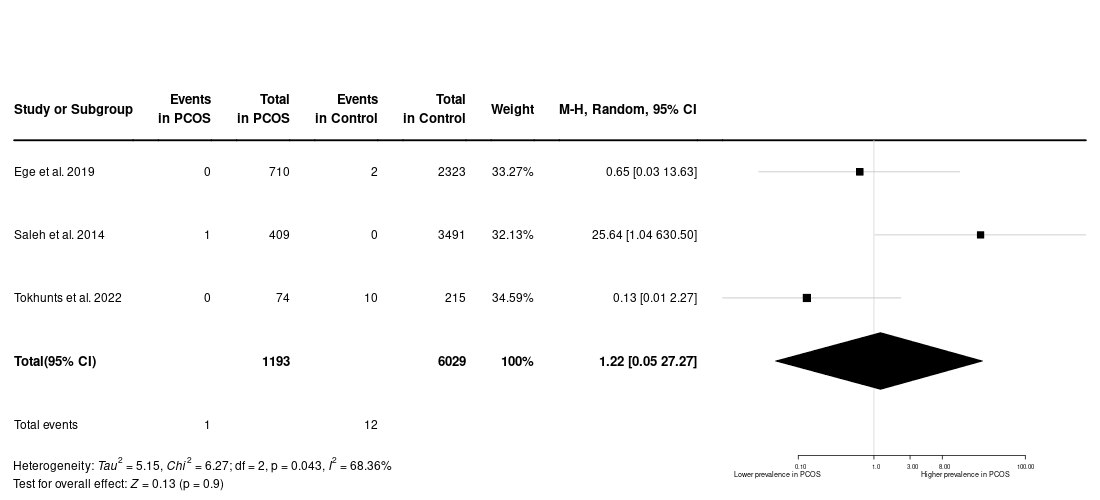
T-shaped uterus
2.
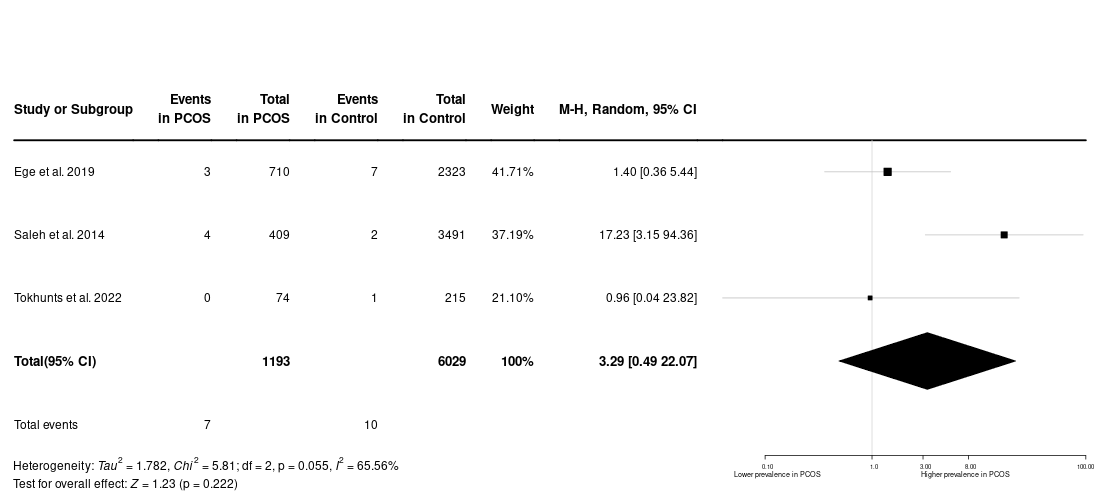
Bicornuate uterus
3.
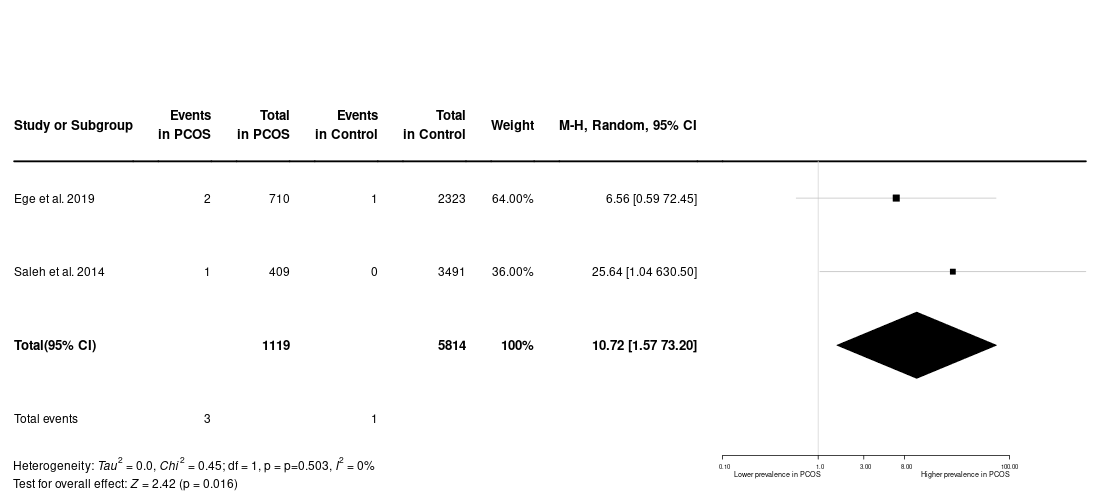
Didelphys uterus
4.
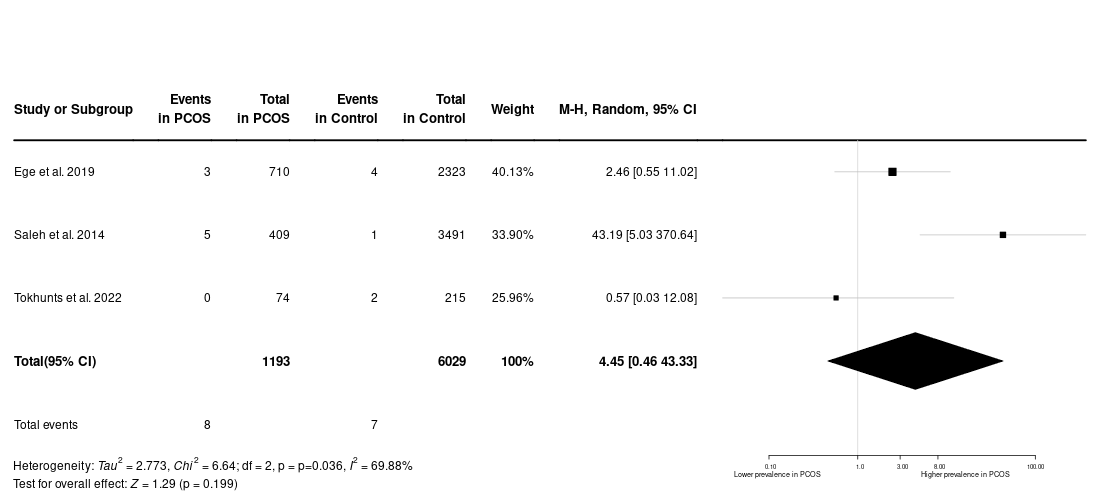
Unicornuate uterus
